# Effective Synthesis of Nucleosides Utilizing *O*-Acetyl-Glycosyl Chlorides as Glycosyl Donors in the Absence of Catalyst: Mechanism Revision and Application to Silyl-Hilbert-Johnson Reaction

**DOI:** 10.3390/molecules22010084

**Published:** 2017-01-05

**Authors:** Chengyuan Liang, Weihui Ju, Shunjun Ding, Han Sun, Gennian Mao

**Affiliations:** Faculty of Pharmacy, Shaanxi University of Science & Technology, 6 Xuefu Road, Xi’an 710021, China; juweihui11521@sina.com (W.J.); 18792439483@163.com (S.D.); hansun0618@163.com (H.S.)

**Keywords:** nucleosides, glycosyl chiorides, *N*-glycosylation, catalyst free reaction, silyl-Hilbert-Johnson reaction

## Abstract

An effective synthesis of nucleosides using glycosyl chlorides as glycosyl donors in the absence of Lewis acid has been developed. Glycosyl chlorides have been shown to be pivotal intermediates in the classical silyl-Hilbert-Johnson reaction. A possible mechanism that differs from the currently accepted mechanism advanced by Vorbrueggen has been proposed and verified by experiments. In practice, this catalyst-free method provides easy access to Capecitabine in high yield.

## 1. Introduction

**Figure 1:**
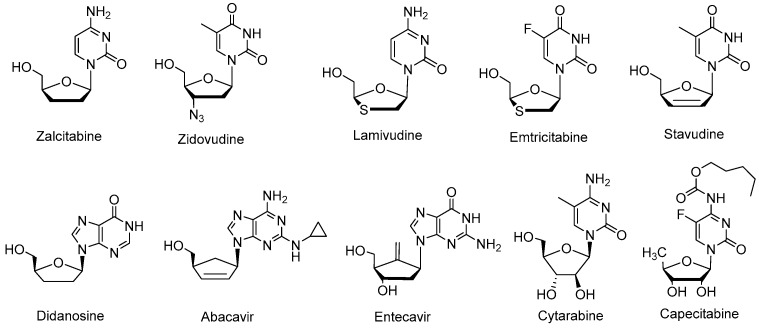
Structures of commonly used nucleoside drugs.

Nucleoside analogs represent a potentially important class of antiviral anticancer agents [1,2,3,4,5] with antimicrobial and cholinesterase inhibitory activities [6,7,8,9,10] and are commonly used to treat hepatitis B virus [11,12], hepatitis C virus [13,14], herpes simplex [15,16], Human Immunodeficiency Virus (HIV) and neoplasms [17,18]. Zalcitabine (2′,3′-dideoxycytidine, ddc), Abacavir, Stavudine (2′,3′-didehydro-2′,3′-didehydro-2′,3′-dideoxythymidine, d4T), Didanosine (2′,3′-dideoxyinosine, ddI), Emtricitabine (FTC) and Zidovudine (azidothymidine, AZT) (Figure 1) are among the essential antiretroviral nucleoside analogue reverse transcriptase inhibitors (NRTIs) that are used to treat HIV/Acquired Immune Deficiency Syndrome (AIDS) infections [19]. In addition, some nucleoside analogues can function as both NRTIs and polymerase inhibitors for other viruses (e.g., Entecavir for hepatitis B) [20]. Less selective nucleoside analogues are used as chemotherapeutic agents to treat cancer, such as the orally administered Capecitabine [21], which is used to treat metastatic breast and colorectal cancers, and Cytarabine [22], which is mainly used in the therapy of white blood cell carcinomas (i.e., acute myeloid leukemia and non-Hodgkin’s lymphoma) (Figure 1). Consequently, the development of new and more effective methods for developing nucleoside analogs is of significant commercial and academic interest.

Well-established strategies of preparing *N*-nucleosides include the following: (1) Fischer and Helferich reported that purine nucleosides could be synthesized by coupling purines with acetobromoglucose and applying silver or mercury salts as catalysts [23,24]; (2) the improved silyl-Hilbert-Johnson reaction [25,26], the most widely used synthetic method, involves the coupling of per-silylated heterocyclic bases with per-acylated sugars in the presence of Friedel-Crafts catalysts (e.g., SnCl_4_ or TMSOTf). This reaction has been the dominant method for the preparation of pyrimidine, purine and other heterocyclic nucleosides. Despite these available methods [8,9,27,28,29,30], a facile and green synthetic strategy, which avoids the use of costly and hazardous catalysts, for synthesizing nucleosides remains an urgent need.

Since the introduction of the silyl-Hilbert-Johnson reaction by Iwai and Nishimura [31], diverse types of glycosyl donors have been applied to prepare nucleosides. Among these donors, 1-*O*-acetyl sugars are the most frequently used glycosyl donors. However, in all of these methods, a stoichiometric amount of a strong Lewis acid promoter (e.g., SnCl_4_, TMSOTf or BF_3_·OEt_2_) is required to secure high-yielding *N*-glycosylation. Notably, 1-Cl-acetyl sugars, inexpensive glycosyl donors, have rarely been employed in nucleoside synthesis, and a catalyst-free *N*-glycosylation method utilizing 1-Cl-acetyl sugars as glycosyl donors has not been reported yet. Herein, we report the facile glycosylation of nucleobases employing 1-Cl-acetyl sugars as glycosyl donors under mild conditions in the absence of any additives.

## 2. Results and Discussion

**Figure 2:**
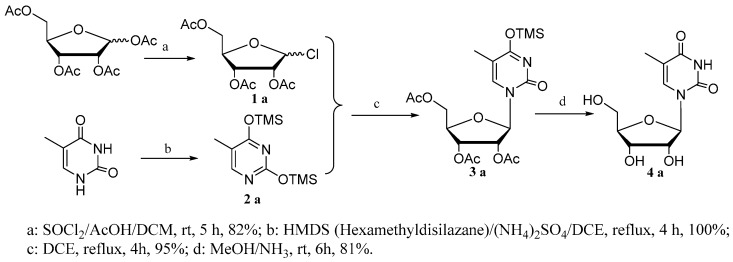
Synthesis of ribosylthymine.

Initially, we attempted to develop an ionic liquid-promoted method of *N*-glycosylation employing peracetylated d-ribofuranosyl chloride (**1a**) as the glycosyl donor. Disappointingly, ionic liquid was unsuitable for the labile silyl-pyrimidine in these preliminary attempts (Entries 6–8, Table 1). A farraginous mixture of products was produced, but no nucleoside was obtained, according to mass spectrometric analysis. In contrast, acetyl nucleoside (**3a**) was obtained with an unexpectedly high yield (95%) when the reaction was carried out in the absence of catalyst (Entry 1, Table 1). The starting material peracetylated d-ribofuranosyl chloride (**1a**) was conveniently prepared by the reaction of per-acylated ribose with stoichiometric thionyl chloride (instead of the commonly used acetyl chloride) in the presence of catalytic glacial acetic acid in dry 1,2-dichloroethane at ambient temperature (Figure 2). Bis-silyl pyrimidine (**2a**) was obtained in a quantitative yield by treating thymine with Hexamethyldisilazane (HMDS) using a catalytic amount of ammonium sulfate in refluxing 1,2-dichloroethane (DCE). Gratifyingly, these important findings encouraged us to further explore the scope of the catalyst-free reaction.

We commenced our study with a brief optimization of the reaction conditions, selecting peracetylated d-ribofuranosyl chloride (**1a**) and per-silylated thymine as starting materials. As shown in Table 1, a trace amount of **3a** was obtained in a polar protic solvent (Entry 5). In contrast, reactions occurring in other solvents (i.e., DCE, MeCN, THF and toluene) at reflux afforded the desired product in moderate to excellent yields (Entries 1–4). As mentioned above, none of the desired product (**3a**) was obtained in ionic liquids (Entries 6–8).

**Figure 3:**
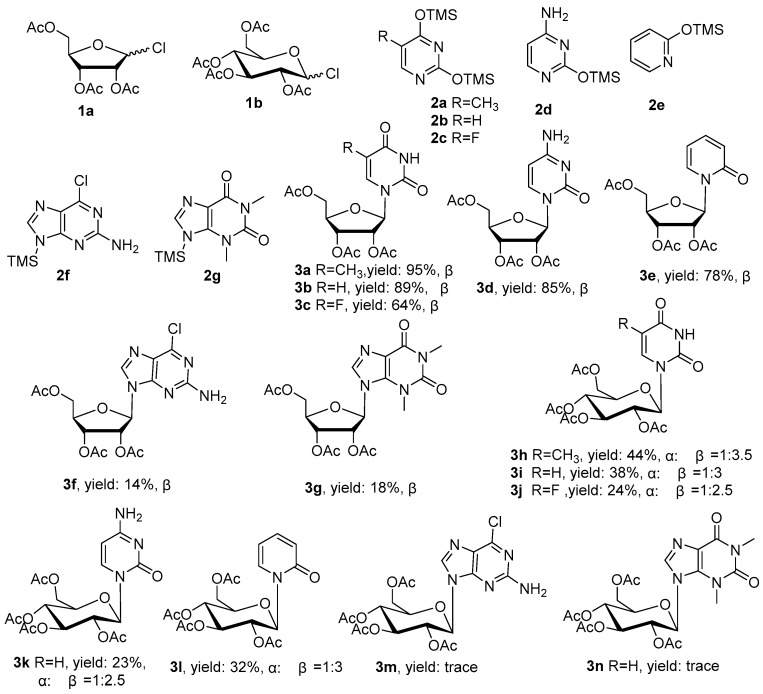
Nucleoside synthesis employing acetyl-glycosyl chlorides as glycosyl donors.

With the optimal reaction conditions in hand, we investigated the reactions of 1-Cl-acetyl sugars **1a** and **1b** with a variety of per-silylated nucleobases (**2a**–**2g**) using 1,2-dichloroethane (DCE) as solvent at 83 °C to establish the generality of the transformation. Compound **1b** was obtained according to the method used to prepare **1a**, and per-silylated nucleobases **2b**–**2g** were prepared in the same manner as **2a**. Reaction completion (after 6–14 h) was determined by thin-layer chromatography (TLC). The results are shown in Figure 3. The glycosylation of per-silylated pyrimidine nucleobases **2a**–**2d** with peracetylated d-ribofuranosyl chloride **1a** afforded the desired β-nucleosides **3a**–**3e** in moderate to excellent yields (64%–95%).The electron-withdrawing group (-F) on silylated 5-fluorouracil led to a decrease in the yield of the *N*-glycosylation reaction (compared to **2a** and **2b**), likely due to the reduced nucleophilicity of **2c**. Purine nucleobases **2f** and **2g** generated the corresponding products **3f** and **3g** in poor yields (14% and 18%), which might have partially been due to difficulties in purification. The coupling of per-silylated pyrimidine nucleobases **2a**–**2e** with tetra-*O*-acetyl glucopyranosyl chloride **1b** led to a mixture of α/β nucleoside stereoisomers **3h**–**3l** in relatively low yields (14%–44%), the ratios determined by the chiral column of HPLC. In addition, **2f** and **2g** failed to generate corresponding products **3m** and **3n**, instead resulting in a complex mixture.

**Figure 4:**
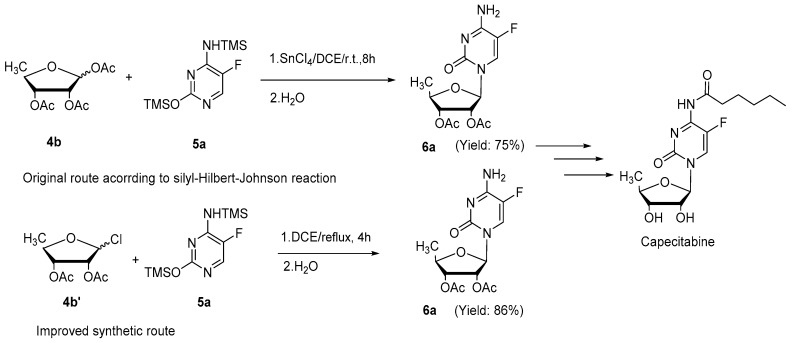
Application to the preparation of Capecitabine.

Perhaps the most striking demonstration of the utility of this reaction is demonstrated by the preparation of Capecitabine (an oral medication for treating breast cancers). As depicted in Figure 4, key intermediate **6a** was obtained employing **4b′** and **5a** as starting materials with improved yield (86%, compared to 75% using a traditional silyl-Hilbert-Johnson reaction) and a significant decrease in the reaction time (4 h compared to 8 h) [32].

**Figure 5:**
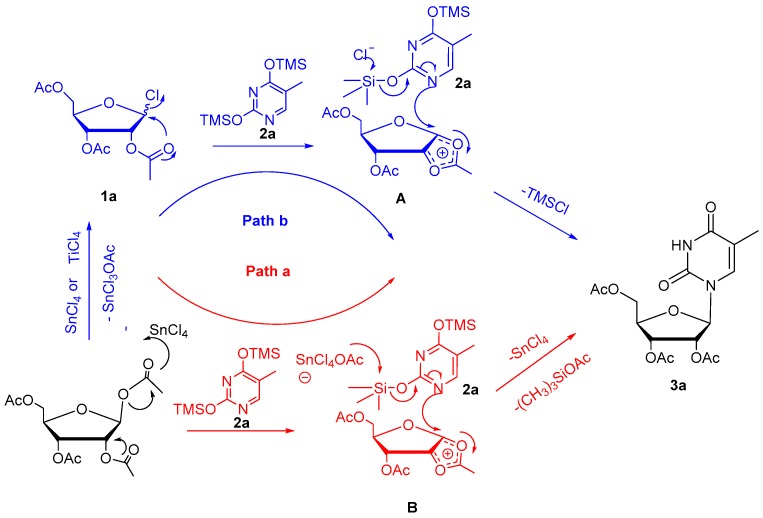
Mechanism for silyl-Hilbert-Johnson reaction.

**Figure 6:**
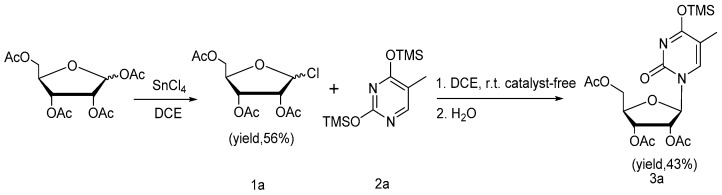
Verification of speculated path b of silyl-Hilbert-Johnson reaction.

Intermediate **A** is quite similar to intermediate **B** formed under silyl-Hilbert-Johnson reaction conditions, according to the classical mechanism (path a in Figure 5) initially proposed by Vorbrueggen [33,34]. Combining this structure with our findings, the efficient and high-yielding reactions described above, indicates that intermediate **A** can be generated smoothly from peracetylated d-ribofuranosyl chloride **1a**. Consequently, we postulate that path **b** (Figure 6) may be an alternative course of the classical mechanism (path a) of the silyl-Hilbert-Johnson reaction. To verify our hypothesis, per-acetyl ribose was treated with SnCl_4_ in the absence of per-silylated nucleobase **2a**, according to silyl-Hilbert-Johnson reaction conditions. As expected, peracetylated d-ribofuranosyl chloride **1a** was obtained in relatively high yield (56%) after stirring for 3 h at ambient temperature. The equivalent stoichiometric silyl-thymidine **2a** without Lewis acid catalyst was added to a solution of peracetylated d-ribofuranosyl chloride **1a** in DCE and the resulting mixture was stirred for 16 h at ambient temperature (Figure 6). Upon completion of the reaction, the crude product **3a** was purified by column chromatography to give a moderate isolated yield of 43%. Therefore, the silyl-Hilbert-Johnson reaction might be characterized by two available routes, path a and path b. The mechanism is shown in Figure 6.

Figure 5 lays out a plausible mechanism for this catalyst-free *N*-glycosylation method. First, **1a** undergoes intramolecular cyclization to give cyclic oxonium ion **A**; this process occurs without any catalyst. Subsequently, **A** reacts with **2a** via an intermolecular nucleophilic substitution reaction to deliver intermediate **B**, which is converted to the target product **3a** after the addition of water. Acidic gas, possibly from the hydrolysis of TMSCl in air, was generated in the reaction, which supports the proposed mechanism.

## 3. Experimental Section

All substrates and solvents were commercially available and were purified before use. Reactions were carried out under N_2_ using standard Schlenk techniques. Mass spectra were recorded on a mass spectrometer using electron impact ionization (EI) techniques. Compounds were visualized under UV lamp (254 nM). ^1^H-NMR and ^13^C-NMR spectra were obtained on a Bruker AV-300 NMR spectrometer (Zurich Region, Switzerland). Analytical TLC was carried out with plates pre-coated with silicagel 60 F_254_ (0.25 mm thick). The identity of the products was established by comparing their physical and spectral data with those of reported compounds [35,36,37,38,39,40,41]. See more details in Supplementary Materials.

### General Experimental Procedure for Synthesis of Acetyl Ribosylthymine *(**3a**)*

Synthesis of **1a**: Ribofuranose tetraacetate (20 mmol) was mixed with thionyl chloride (12 mmol) and glacial acetic acid (0.1 mmol) under stirring in 1,2-dichloroethane (50 mL) for 6 h at room temperature. The obtained product was purified by column chromatography (MeOH/CH_2_Cl_2_, 1:10 *v*/*v*). Compound **1b** was prepared under the same conditions with a longer reaction time (10 h).

Synthesis of **2a**: A mixture of thymidine (10 mmol), HMDS (12 mmol) and ammonium sulphate (0.1 mmol) was stirred in refluxing 1,2-dichloroethane (50 mL) for 3 h. When the reaction mixture became translucent, it was concentrated in vacuo to provide **2a**. Compounds **2b**–**f** were prepared under the same conditions with varying reaction times (4–8 h).

Synthesis of **3a**: To a solution of silyl-thymidine (10 mmol) in DCE (100 mL) was added **1a** (12 mmol diluted in 25 mL DCE) dropwise at room temperature. The resulting mixture was stirred for 4 h at 83 °C. Upon completion of the reaction, as indicated by TLC (*n*-hexane/ethylacetate: 40:60), the reaction mixture was cooled to room temperature and H_2_O (50 mL) was added, and the mixture was, stirred for 0.5 h. The resulting reaction mixture was diluted with DCE (50 mL) and washed with water and saturated NaCl solution. The organic layer was dried with MgSO_4_ and filtered. The filtrate was concentrated under reduced pressure and purified by column chromatography (MeOH/CH_2_Cl_2_, 1:10 *v*/*v*) to obtain an analytical sample for a total yield of 95%.

## 4. Conclusions

In summary, we have developed an efficient and straightforward synthesis of N-nucleosides employing glycosyl chlorides as glycosyl donors in the absence of traditional Lewis acid catalysts, which significantly expands the versatility of the silyl-Hilbert-Johnson reaction. The higher yields, mild reaction conditions, ease of purification, economic availability of the starting materials and avoidance of a catalyst make this an ecologically friendly procedure for the synthesis of nucleosides. A novel dual-path mechanism for the silyl-Hilbert-Johnson reaction has also been proposed and validated experimentally. In addition to Capecitabine, further expansions of the reaction scope and synthetic applications of this methodology are in progress in our laboratory.

## Figures and Tables

**Table 1:** Optimization of reaction conditions for the synthesis of **3a**.

Entry	Solvent	Reaction Time (h)	Temprature (°C)	Yield (%)
1	1,2-Dichloroethane	4	83	95
2	Toluene	6	110	89
3	MeCN	7	80	78
4	THF	10	66	64
5	Ethanol	10	78	Trace
6	[Bmim]BF_4_	8	90	-
7	[Bmim]PF_6_	8	90	-
8	[Bmim]HSO_4_	8	90	-

## Supplementary Materials

Supplementary materials can be accessed at: http://www.mdpi.com/1420-3049/22/1/84/s1.
