# Supplementary material for: Effective Synthesis of Nucleosides Utilizing O-Acetyl-Glycosyl Chlorides as Glycosyl Donors in the Absence of Catalyst: Mechanism Revision and Application to Silyl-Hilbert-Johnson Reaction
Source: Molecules. 2017 Jan 5;22(1):84. doi: 10.3390/molecules22010084 (PMC6155650; doi:10.3390/molecules22010084)

Supplementary Materials: Effective Synthesis of Nucleosides Utilizing Acetyl-Glycosyl Chlorides as Glycosyl Donors in the Absence of Catalyst: Mechanism Revision and Application to Silyl-Hilbert-Johnson Reaction

Chengyuan Liang, Weihui Ju, Shunjun Ding, Han Sun and Gennian Mao

1. General

All substrates and solvents were commercially available and were purified before use. Reactions were carried out under N_2 using standard Schlenk techniques. Mass spectra were recorded on a mass spectrometer using electron impact ionization (EI) techniques. Compounds were visualized under UV lamp (254 nM). Column chromatography was performed on 100–200 mesh silica gel. Bruker Avance 300 MHz and Bruker Avance 75 MHz spectrometer was used to record for ^{1}H- and ^{13}C-NMR respectively, using tetramethylsilane (δ = 0.00) as an internal standard at 25 °C. ^{1}H- and ^{13}C-NMR spectra were also obtained on a Bruker Avance III 400 MHz NMR spectrometer. Mass spectra were recorded on a mass spectrometer using electron impact ionization (EI) techniques. Analytical TLC was carried out with plates pre-coated with silicagel 60 F_{254} (0.25 mm thick). The identity of the products was established by comparing their physical and spectral data with those of reported compounds.

2. Analysis Data

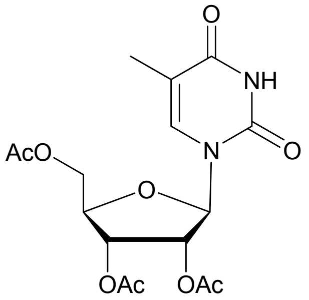

3a

3a: *(2R,3R,4R,5R)-2-(acetoxymethyl)-5-(5-methyl-2,4-dioxo-3,4-dihydropyrimidin-1(2H)-yl)tetrahydrofuran-3,4-diyldiacetate*. ^{1}H-NMR (300 MHz, $CDCl_3$, 303 K) δ: 8.937 (1H, s), 7.102 (1H, d, *J* = 7.5 Hz), 6.088 (2H, d, *J* = 7.5 Hz), 5.462 (1H, d, *J* = 7.8 Hz), 5.362 (1H, d, *J* = 7.6 Hz), 4.358 (1H, s), 4.228 (1H, s), 2.346–2.079 (12H, m); ^{13}C-NMR (75 MHz, $CDCl_3$, 303 K) δ: 170.259, 163.775, 150.459, 136.571, 110.309, 96.571, 81.608, 71.608, 64.966, 21.608, 20.453, 9.356; HRMS (ESI) for $(M + Na)^+$: calcd. 407.3380, found 407.3382.

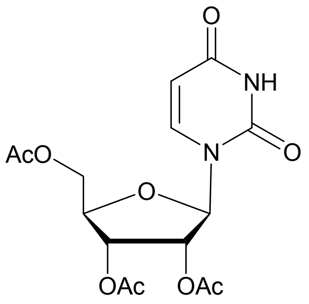

3b

3b: *(2R,3R,4R,5R)-2-(acetoxymethyl)-5-(2,4-dioxo-3,4-dihydropyrimidin-1(2H)-yl)tetrahydrofuran-3,4-diyldiacetate.* ^{1}H-NMR (300 MHz, $CDCl_3$, 303 K) δ: 8.782 (1H, s), 7.402 (1H, d, *J* = 7.4 Hz), 6.048 (1H, d), 5.816 (1H, d, *J* = 7.4 Hz), 5.346 (2H, d, *J* = 7.6 Hz), 4.324 (3H, m, *J* = 7.4 Hz), 2.099–2.066(9H, m); ^{13}C-NMR (75 MHz, DMSO-d_6, 303 K) δ: 170.188, 163.303, 150.803, 140.200, 102.403, 96.898, 81.808, 75.336, 62.209, 20.898; HRMS (ESI) for $(M + H)^+$: calcd. 371.6114 , found 371.6115.

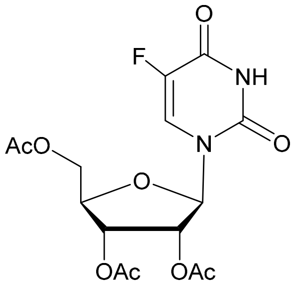

3c

3c: *(2R,3R,4R,5R)-2-(acetoxymethyl)-5-(5-fluoro-2,4-dioxo-3,4-dihydropyrimidin-1(2H)-yl)tetrahydrofuran-3,4-diyldiacetate.* ^{1}H-NMR (300 MHz, $CDCl_3$, 303 K) δ: 10.005 (1H, s), 8.105 (1H, s), 6.468 (1H, d, *J* = 7.5 Hz), 6.298 (1H, d, *J* = 7.5 Hz), 5.575–5.346 (2H, m, *J* = 7.4 Hz), 4.364–4.250 (2H, m, *J* = 7.8 Hz), 2.096–2.041 (9H, m); ^{13}C-NMR (75 MHz, DMSO-d_6) δ: 170.188, 158.303, 150.803, 140.200, 128.803, 96.898, 81.808, 75.336, 62.209, 20.898; HRMS (ESI) for $(M + Na)^+$: calcd. 411.1109, found 411.1121.

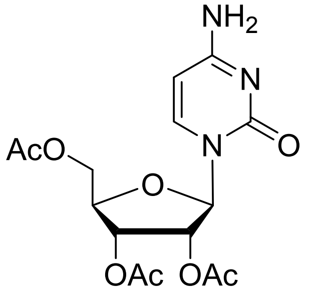

3d

3d: *(2R,3R,4R,5R)-2-(acetoxymethyl)-5-(4-amino-2-oxopyrimidin-1(2H)-yl)tetrahydrofuran-3,4-diyldiacetate.* ^{1}H-NMR (300 MHz, $CDCl_3$, 303 K) δ: 8.059 (1H, s), 6.785 (2H, s), 6.325 (1H, d), 6.285 (1H, d), 5.342–4.902 (3H, m, *J* = 7.8 Hz), 4.339–4.018 (2H, m, *J* = 7.6 Hz), 2.131–2.059 (9H, m); ^{13}C-NMR (75 MHz, DMSO-d_6, 303 K) δ: 170.369, 165.337, 155.497, 143.535, 95.995, 94.020, 81.990, 75.525, 74.073, 62.243, 21.382; HRMS (ESI) for $(M + H)^+$: calcd. 370.2413 , found 370.2415.

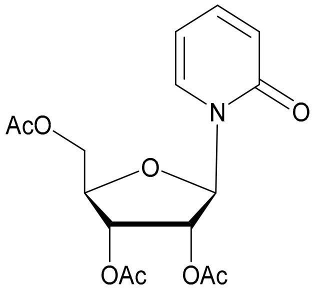

3e

3e: *(2R,3R,4R,5R)-2-(acetoxymethyl)-5-(2-oxopyridin-1(2H)-yl)tetrahydrofuran-3,4-diyldiacetate.* ^{1}H-NMR (300 MHz, $CDCl_3$, 303 K) δ: 8.030 (1H, s), 7.507 (1H, m, *J* = 7.3 Hz), 7.093 (1H, d), 6.493 (2H, m, *J* = 7.3

Hz), 5.161 (1H, t, *J* = 7.6 Hz), 4.972–4.925 (2H, m, *J* = 7.6 Hz), 3.761–3.581 (2H, m, *J* = 7.8 Hz), 2.020–1.835 (9H, m); ^{13}C-NMR (75 MHz, $CDCl_3$, 303 K) δ: 170.937, 160.948, 140.936, 140.635, 134.955, 106.605, 94.241, 82.159, 70.936, 70.848, 62.506, 20.828, 20.605; HRMS (ESI) for $(M + H)^+$: calcd. 354.3240, found 354.3242.

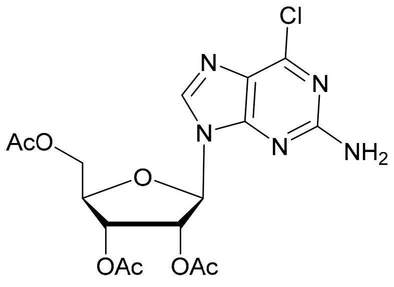

3f

3f: *(2R,3S,4S,5R)-2-(acetoxymethyl)-5-(2-amino-6-chloro-9H-purin-9-yl)tetrahydrofuran-3,4-diyldiacetate.* ^{1}H-NMR (300 MHz, $CDCl_3$, 303 K) δ: 8.120(1H, s), 6.242 (2H, s), 5.342–5.218 (2H, m, *J* = 7.3 Hz), 4.902 (1H, d, *J* = 7.4 Hz), 4.380–4.080 (2H, m, *J* = 7.4 Hz), 2.171–2.059 (9H, m). ^{13}C-NMR (75 MHz, $CDCl_3$, 303 K) δ: 170.084, 159.684, 148.024, 148.004, 131.565, 95.491, 81.540, 71.455, 72.5, 20.307; HRMS (ESI) for $(M + Na)^+$: calcd. 450.7964, found 450.7966.

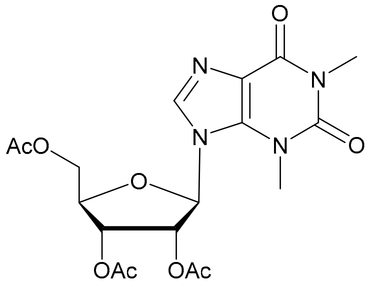

3g

3g: *(2R,3R,4R,5R)-2-(acetoxymethyl)-5-(1,3-dimethyl-2,6-dioxo-1,2,3,6-tetrahydro-9H-purin-9-yl)tetrahydrofuran-3,4-diyldiacetate.* ^{1}H-NMR (300 MHz, $CDCl_3$, 303 K) δ: 8.120 (1H, s), 6.780 (1H, d, *J* = 8.1 Hz), 5.342–5.218 (2H, m, *J* = 7.4 Hz), 4.902 (1H, s), 4.389–4.080 (2H, m, *J* = 8.1 Hz), 3.449–3.349 (6H, m, *J* = 7.4 Hz), 2.136–2.059 (9H, m); ^{13}C-NMR (75 MHz, DMSO-d_6, 303 K) δ: 170.627, 151.585, 150.538, 135.038, 107.238, 88.208, 80.223, 73.181, 72.203, 62.039, 29.939, 28.935, 19.981, 19.039; HRMS (ESI) for $(M + H)^+$: calcd. 439.3887, found 439.3889.

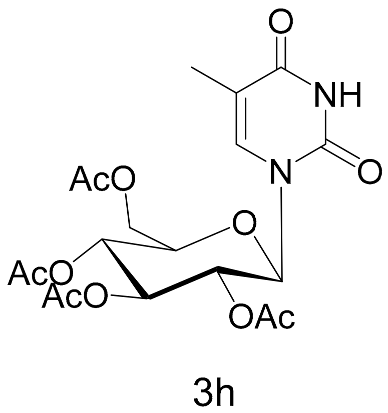

3h

3h: *(2R,3R,4S,5R,6R)-2-(acetoxymethyl)-6-(5-methyl-2,4-dioxo-3,4-dihydropyrimidin-1(2H)-yl)tetrahydro-2H-pyran-3,4,5-triyl triacetate*. ^{1}H-NMR (300 MHz, $CDCl_3$, 303 K) δ: 8.336(1H, s), 7.118 (1H, d, *J* = 1.2 Hz), 5.841 (1H, d, *J* = 8.9 Hz), 5.541–5.520 (2H, m, *J* = 7.9 Hz), 4.192–4.055 (2H, m), 2.094–1.697 (15H, m); ^{13}C-NMR (75 MHz, DMSO-d_6, 303 K) δ: 170.627, 162.585, 150.538, 110.238, 85.208, 75.223, 66.123, 63.439, 62.039, 20.519. 19.039, 14.123; HRMS (ESI) for $(M + H)^+$: calcd. 457.4007, found 457.4010.

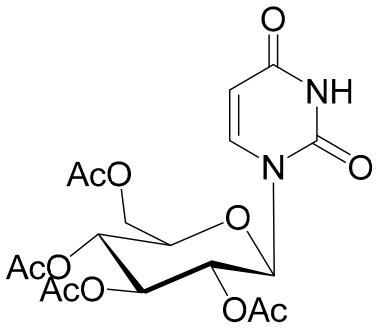

3i

3i: *(2R,3R,4S,5R,6R)-2-(acetoxymethyl)-6-(2,4-dioxo-3,4-dihydropyrimidin-1(2H)-yl)tetrahydro-2H-pyran-3,4,5-triyl triacetate*. ^{1}H-NMR (300 MHz, $CDCl_3$, 303 K) δ: 9.161 (1H, s), 9.396 (1H, d), 7.412 (1H, d, *J* = 8.2 Hz), 5.831 (2H, d), 5.581 (1H, s), 5.283–2.208 (2H, m, *J* = 8.1 Hz), 4.280–4.158 (3H, m, *J* = 7.5 Hz), 2.139–2.007 (12H, m); ^{13}C-NMR (75 MHz, DMSO-d_6, 303 K) δ: 170.445, 170.133, 170.016, 163.045, 150.845, 140.736, 102.436, 86.261, 72.223, 71.942, 69.161, 68.515, 20.774, 20.382; HRMS (ESI) for $(M + Na)^+$: calcd. 465.3741, found 465.3743.

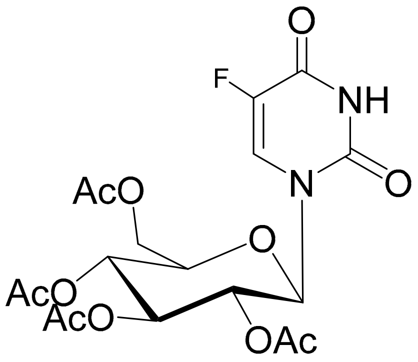

3j

3j: *(2R,3R,4S,5R,6R)-2-(acetoxymethyl)-6-(5-fluoro-2,4-dioxo-3,4-dihydropyrimidin-1(2H)-yl)tetrahydro-2H-pyran-3,4,5-triyl triacetate*. ^{1}H-NMR (300 MHz, $CDCl_3$, 303 K) δ: 10.581 (1H, s), 7.604 (1H, d), 6.117 (1H, d), 5.313–5.248 (2H, m), 4.380–4.117 (4H, m, *J* = 7.8 Hz), 2139–2.007 (12H, m); ^{13}C-NMR (75 MHz, DMSO-d_6, 303 K) δ: 170.434, 158.124, 150.372, 140.407, 127.937, 86.209, 74.327, 67.084, 66.962, 64.089, 63.609, 20.104, 20.099; HRMS (ESI) for $(M + Na)^+$: calcd. 483.3645, found 483.3647.

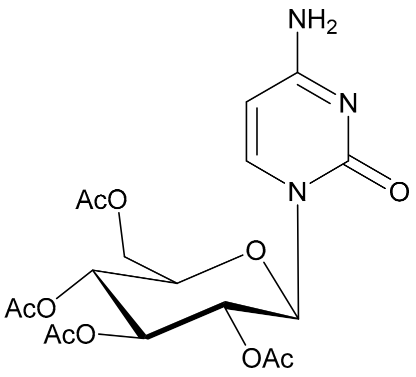

3k

3k: *(2R,3R,4S,5R,6R)-2-(acetoxymethyl)-6-(4-amino-2-oxopyrimidin-1(2H)-yl)tetrahydro-2H-pyran-3,4,5-triyl triacetate*. ^{1}H-NMR (300 MHz, $CDCl_3$, 303 K) δ: 8.032 (1H, d), 6.361 (2H, s), 6.144 (1H, d), 6.036 (2H, d, *J* = 7.8 Hz), 5.352 (1H, s), 5.159 (1H, d), 4.275–3.926 (2H, m, *J* = 7.8 Hz), 2.016–1.861 (12H, m); ^{13}C-NMR (75 MHz, DMSO-d_6, 303 K) δ: 170.369, 165.337, 155.497, 143.535, 94.020, 90.995, 90.673, 87.523, 65.525, 64.073, 15.382; HRMS (ESI) for $(M + Na)^+$: calcd. 464.3893, found 464.3899.

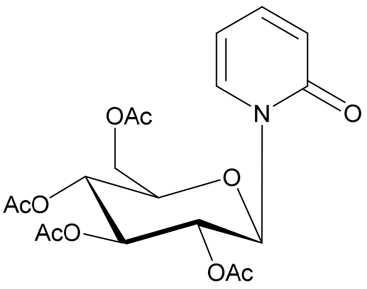

3l

3l: *(2R,3R,4S,5R,6R)-2-(acetoxymethyl)-6-(2-oxopyridin-1(2H)-yl)tetrahydro-2H-pyran-3,4,5-triyl triacetate.* ^{1}H-NMR (300 MHz, $CDCl_3$, 303 K) δ: 8.271 (1H, d), 8.163 (1H, d), 7.745–7.716 (1H, t), 6.161 (1H, s), 6.091 (1H, s), 5.330–5.013 (4H, m, *J* = 7.9 Hz), 4.331–4.050 (2H, m, *J* = 8.0 Hz), 2.176–1.975 (12H, m); ^{13}C-NMR (75 MHz, DMSO-d_6, 303 K) δ: 170.260, 160.721, 139.384, 137.023, 132.421, 110.721, 102.260, 91.319, 77.052, 63.575, 62.887, 62.044, 21.574, 20.863; HRMS (ESI) for $(M + H)^+$: calcd. 426.3866, found 426.3867.

4. Capecitabine

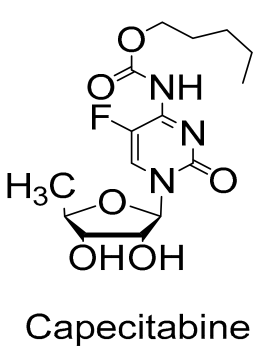

Capecitabine

^{1}H-NMR (300 MHz, DMSO-d_6, 303 K) δ: 8.04(1H, s), 5.66(1H, d), 5.42 (1H, m, *J* = 7.5 Hz), 5.06 (1H, m, *J* = 7.5 Hz), 4.07 (1H, m), 3.82 (1H, m, *J* = 7.4 Hz), 3.66 (2H, m, *J* = 7.4 Hz), 3.49 (2H, s), 2.50 (2H, m), 1.65 (4H, m), 1.32 (3H, m), 0.9 (3H, m); ^{13}C-NMR (75 MHz, DMSO-d_6, 303 K) δ: 160.60, 155.17, 154.04,

126.02, 100.63, 99.20, 85.28, 79.20, 74.04, 65.38, 27.85, 27.39, 21.73, 18.23, 13.82; HRMS (ESI) for (M + H)$^{+}$: calcd. 360.2501, found 360.2504.

5. Hydrogen Spectrum

5.1. H¹-NMR of **3a**

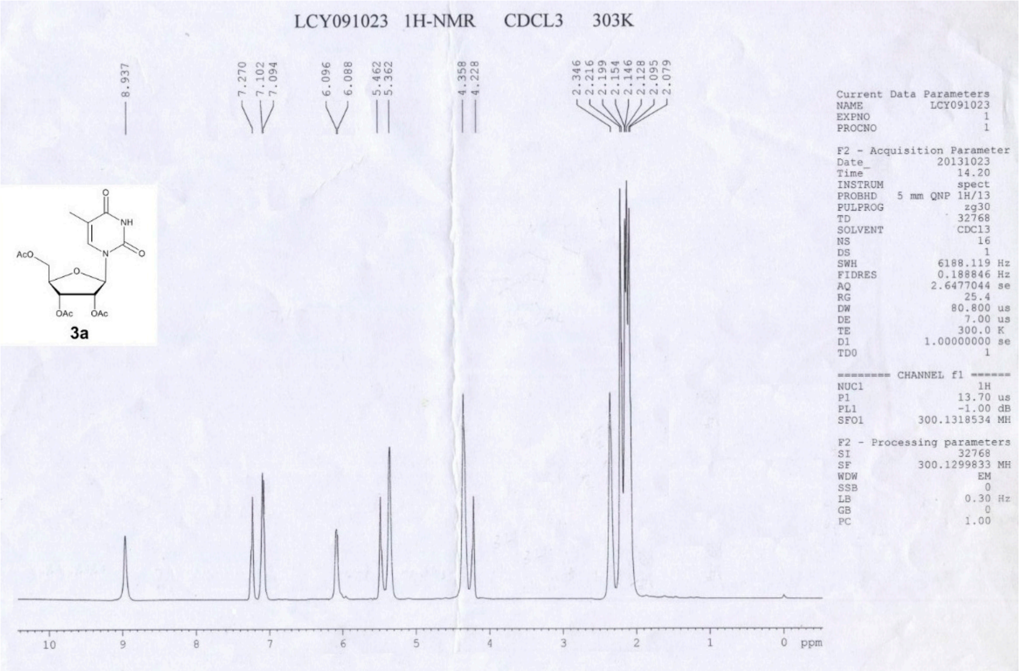

5.2. H¹-NMR of **3b**

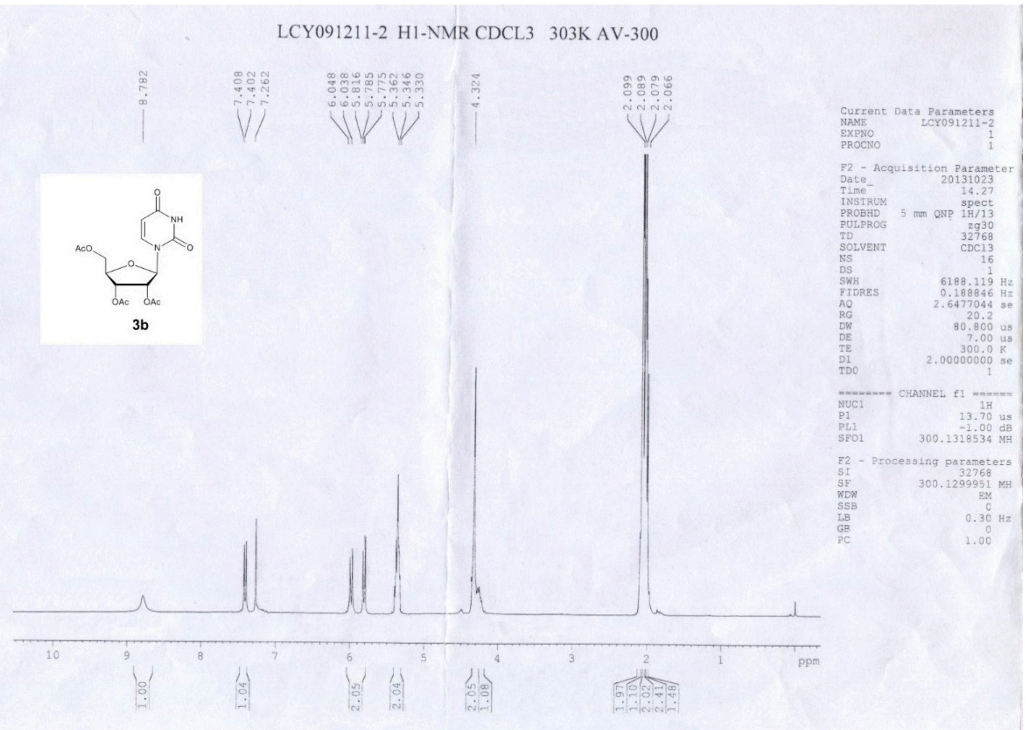

5.3. *H*[1]*-NMR of* **3c**

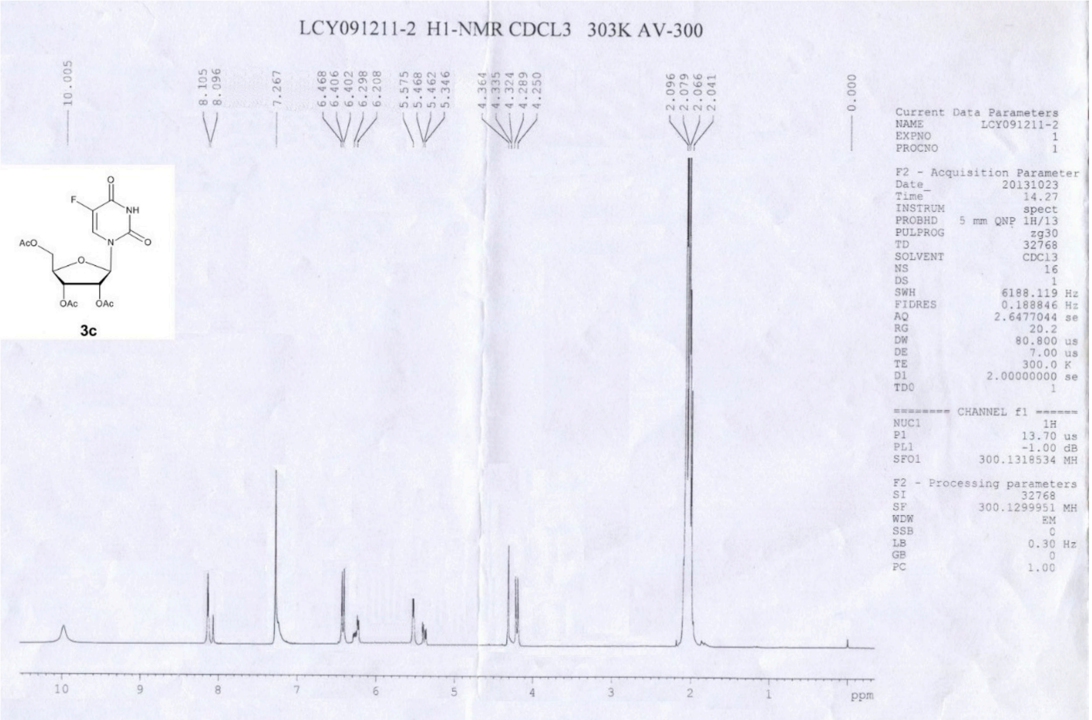

5.4. *H*[1]*-NMR of* **3d**

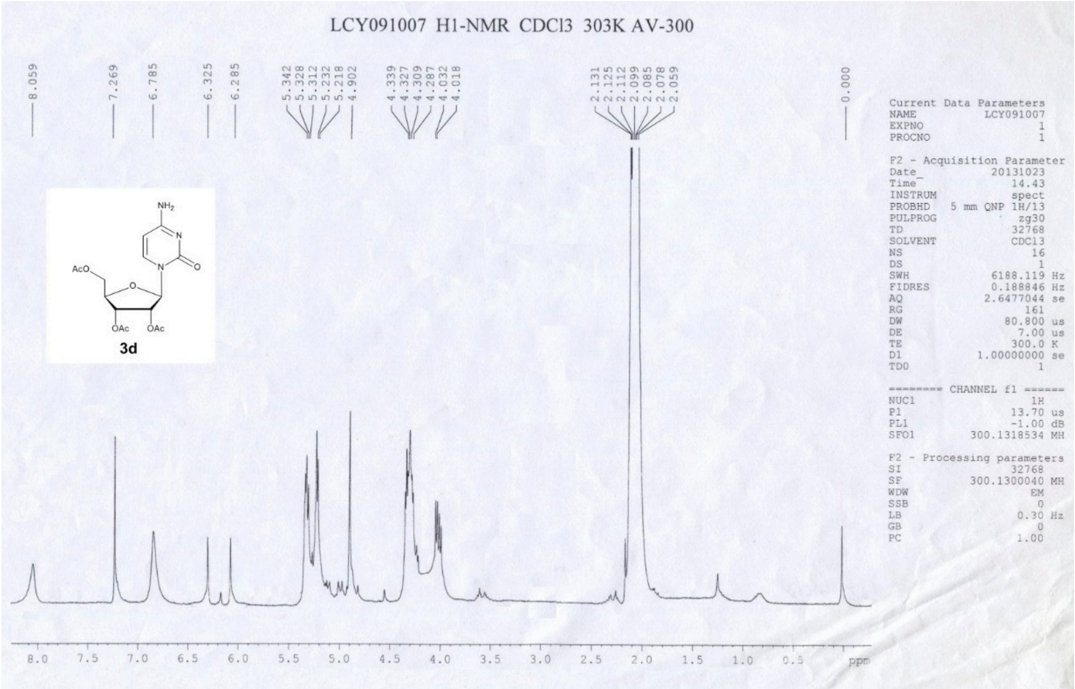

5.5. H¹-NMR of **3e**

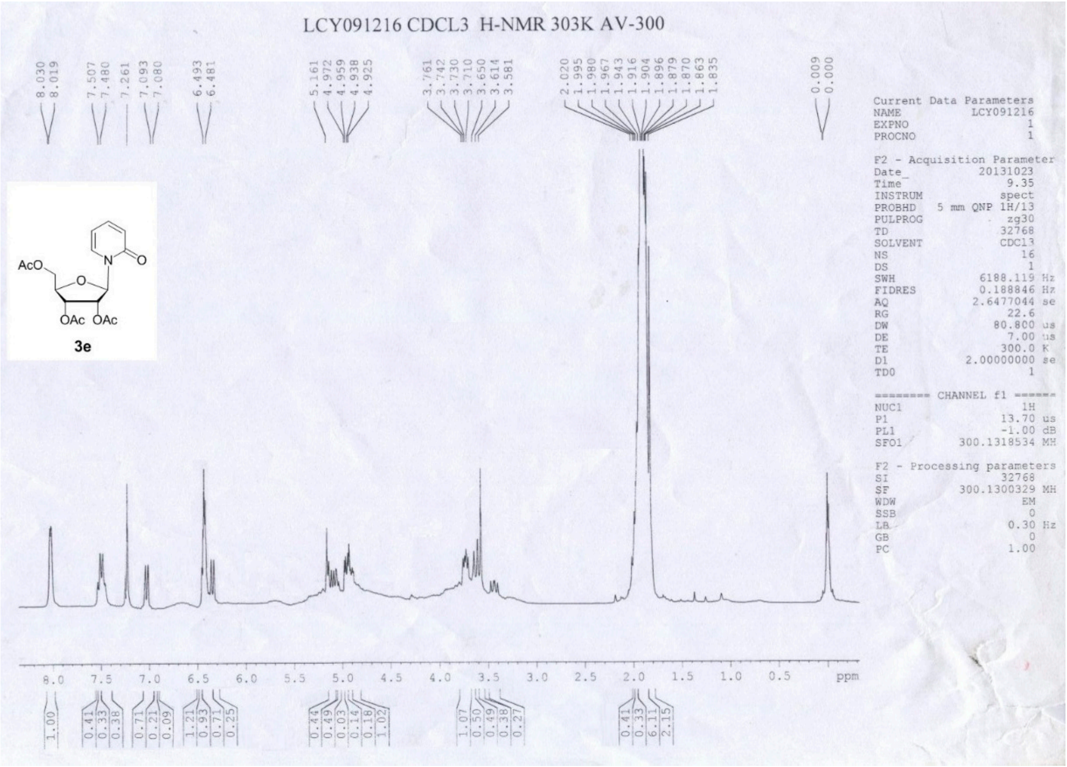

5.6. H¹-NMR of **3f**

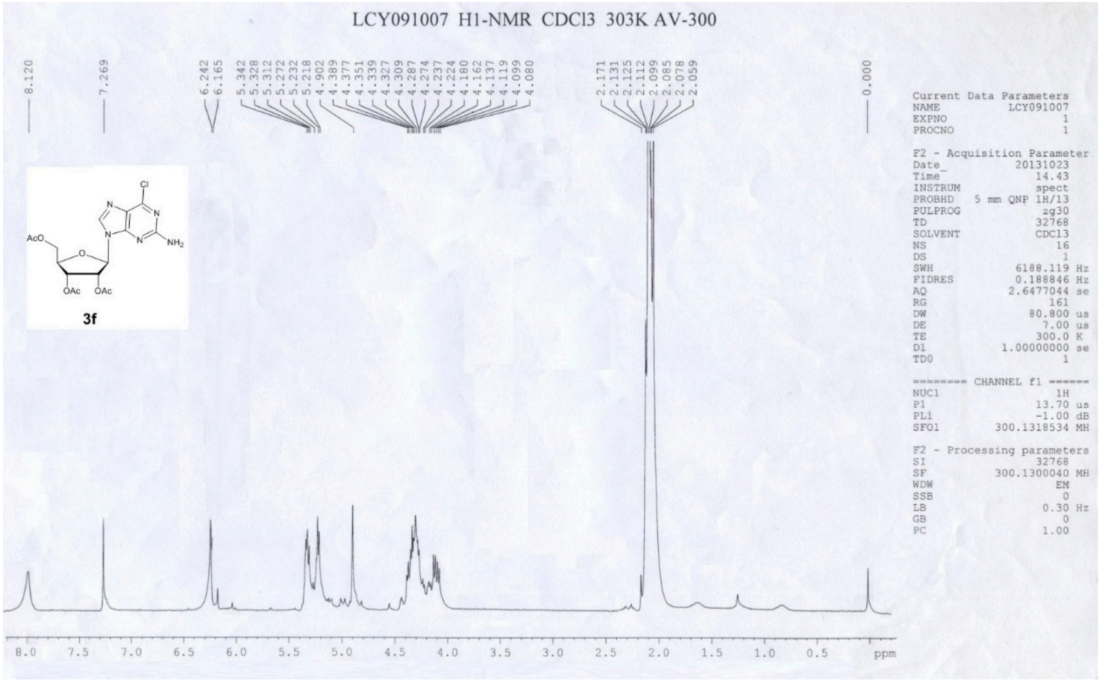

5.7. *H*[1]*-NMR of* **3g**

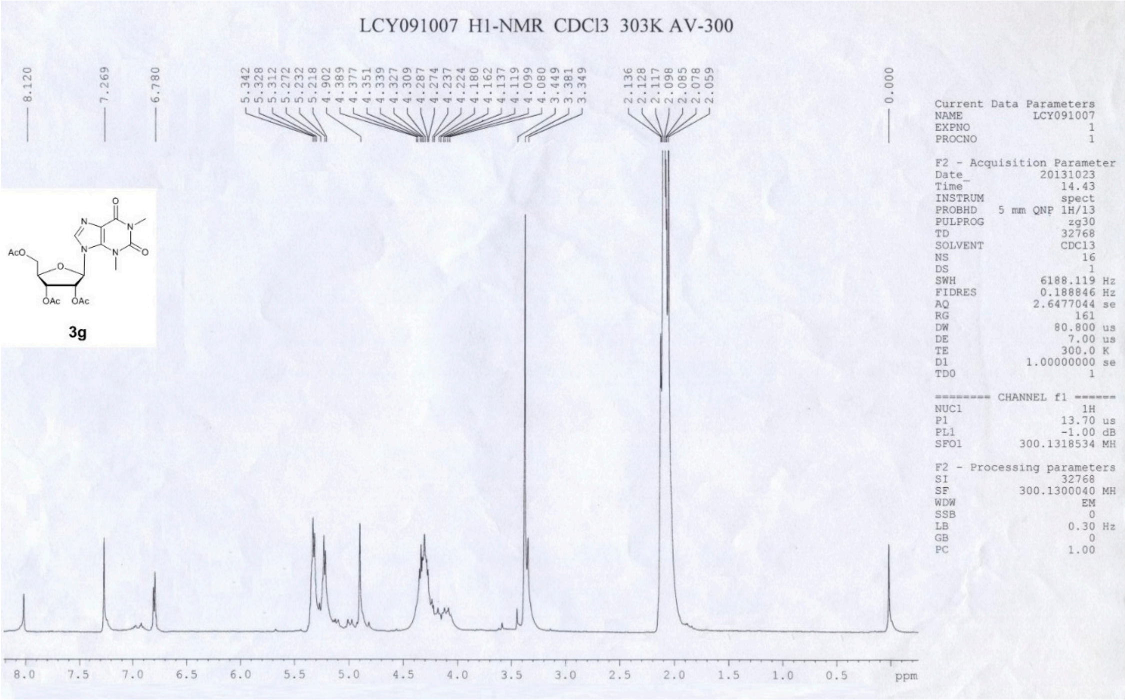

5.8. *H*[1]*-NMR of* **3h**

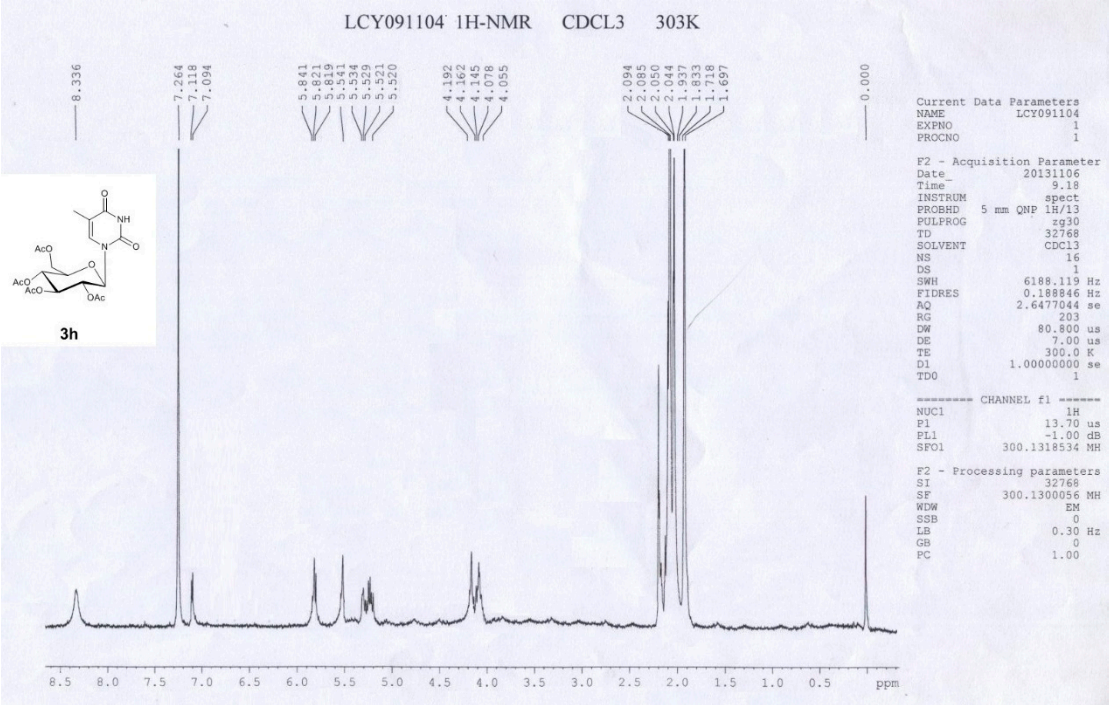

5.9. H¹-NMR of **3l**

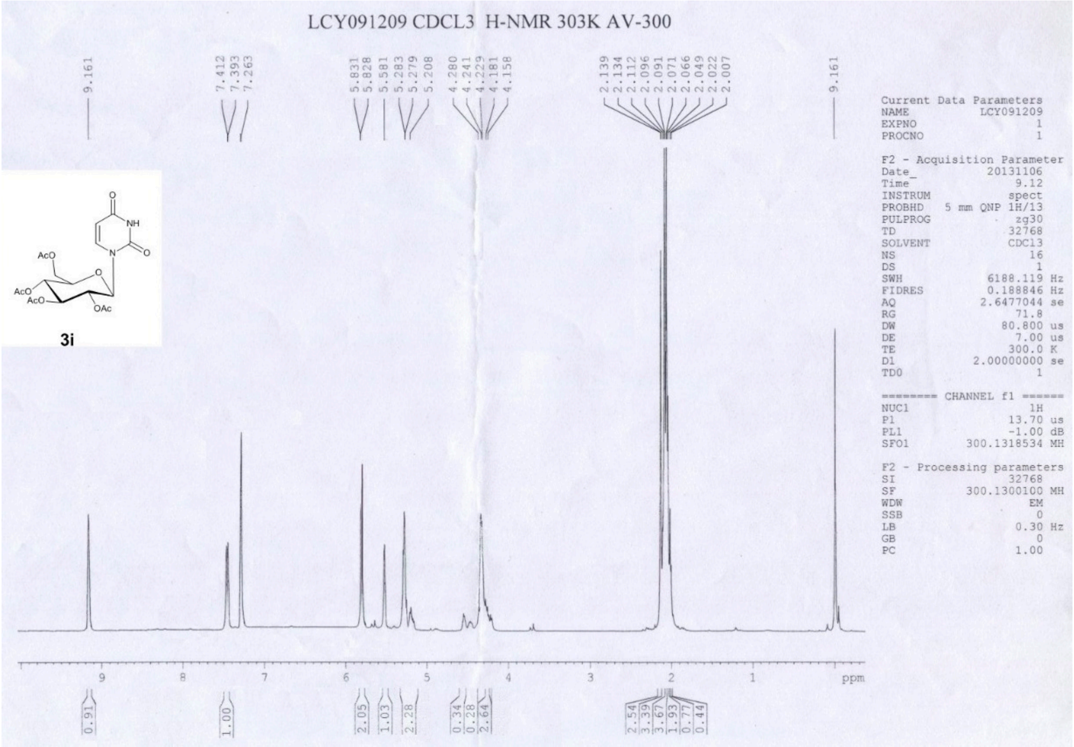

5.10. H¹-NMR of **3j**

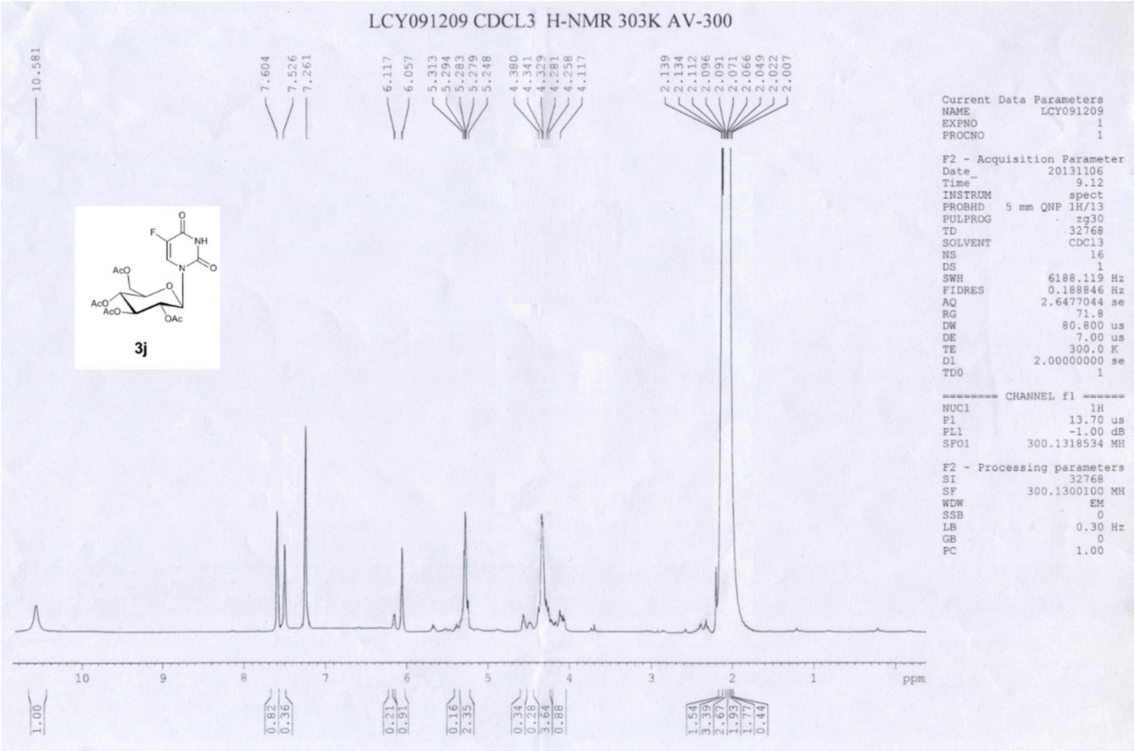

5.11. H¹-NMR of **3k**

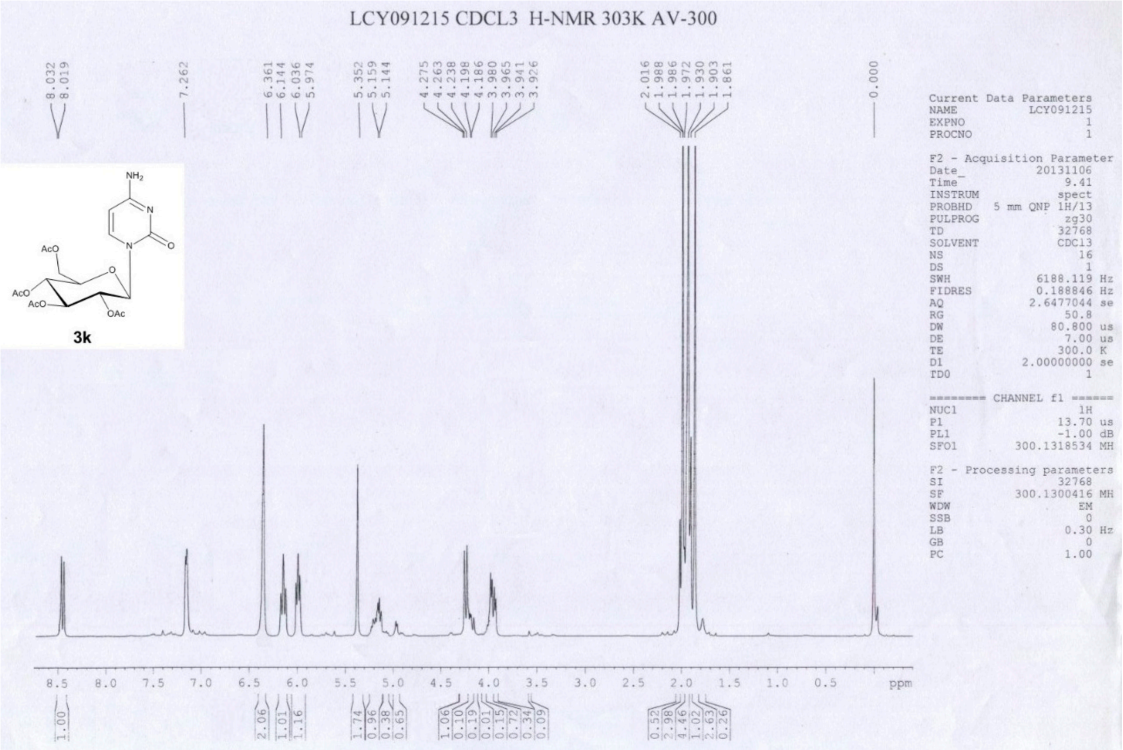

5.12. H¹-NMR of **3l**

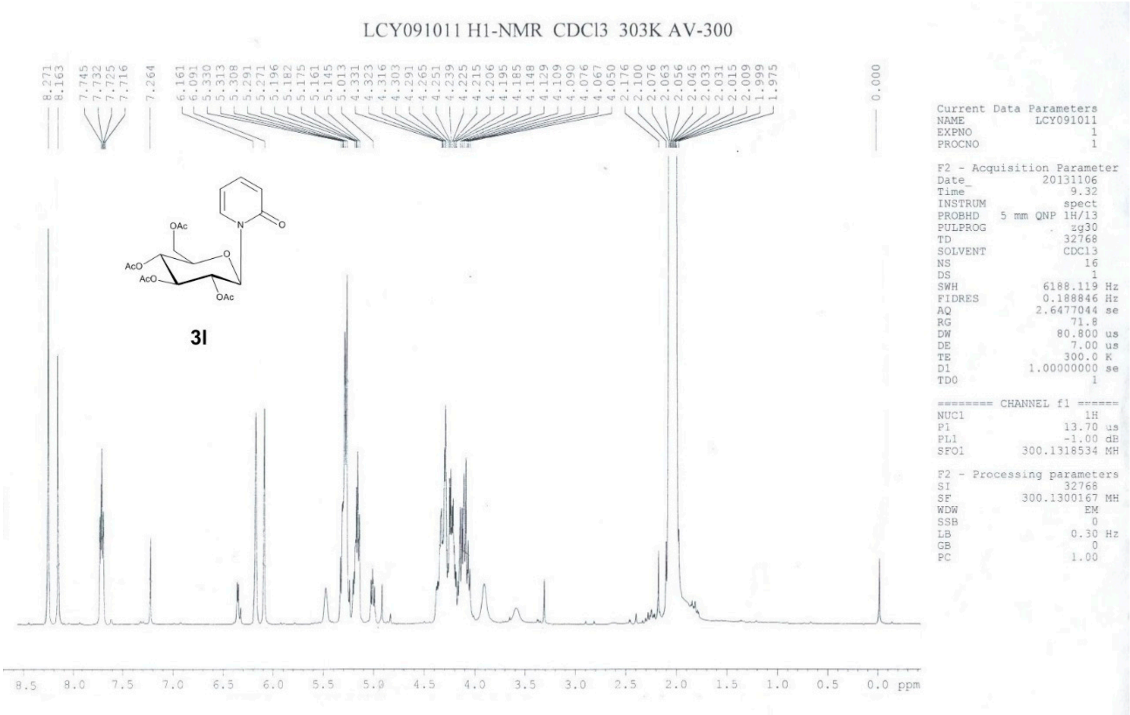

5.13. H[1]-NMR of Capecitabine

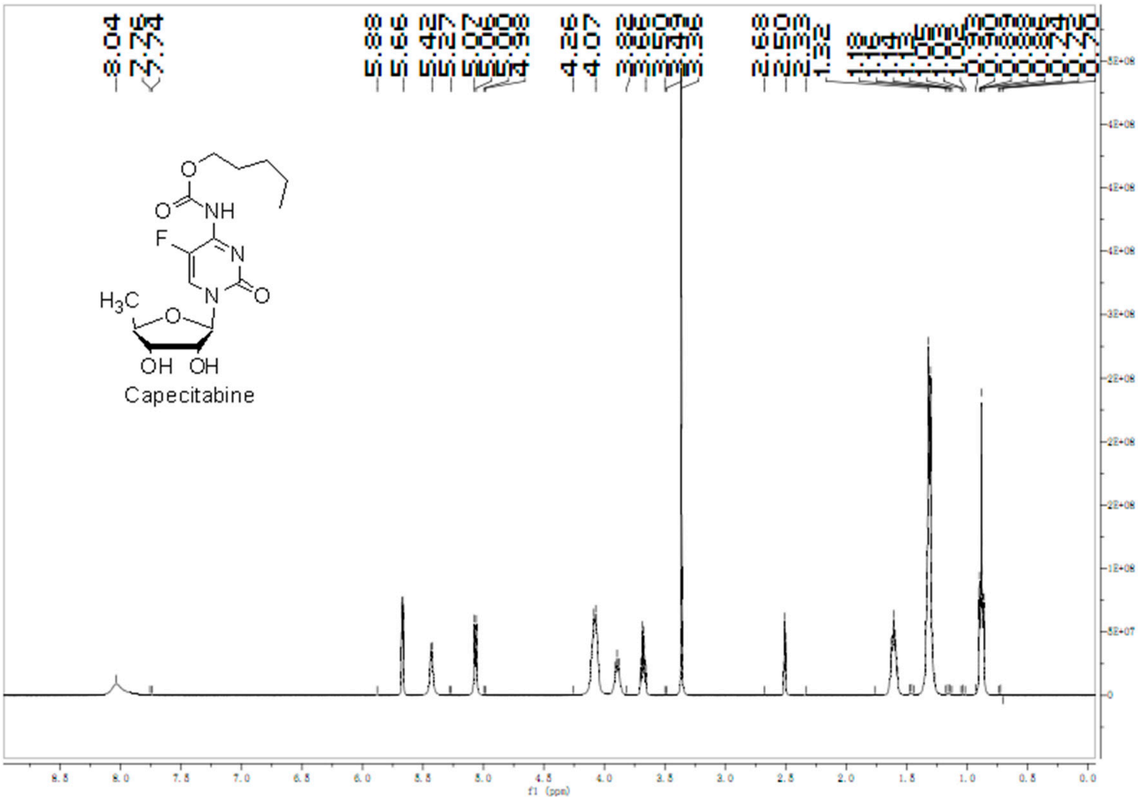

6. Carbon Spectrum

6.1. C[13]-NMR of **3a**

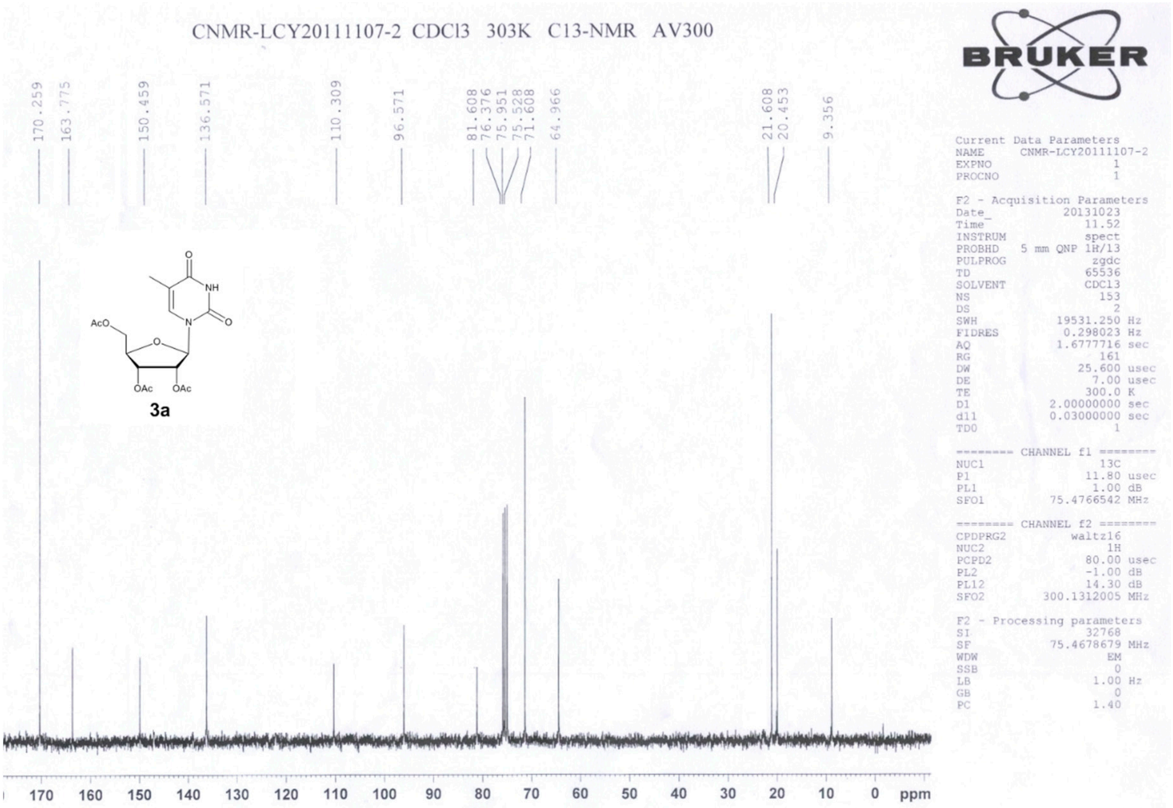

6.2. C13-NMR of **3b**

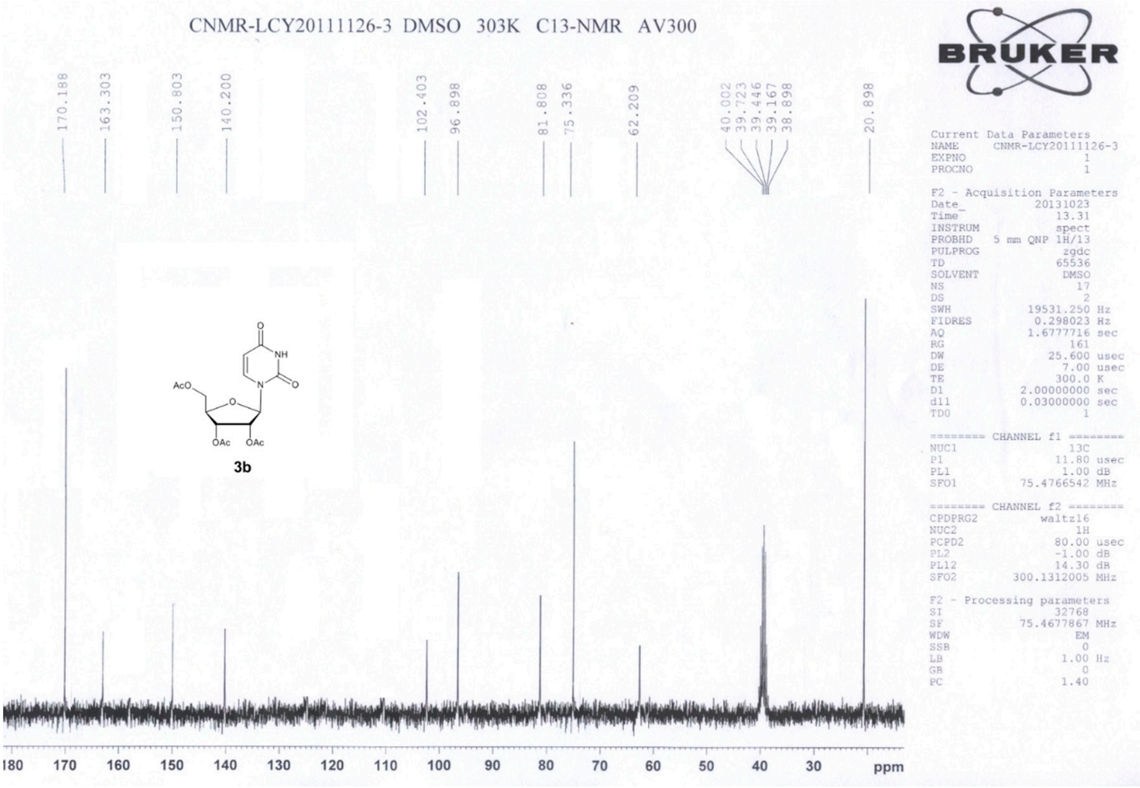

6.3. C13-NMR of **3c**

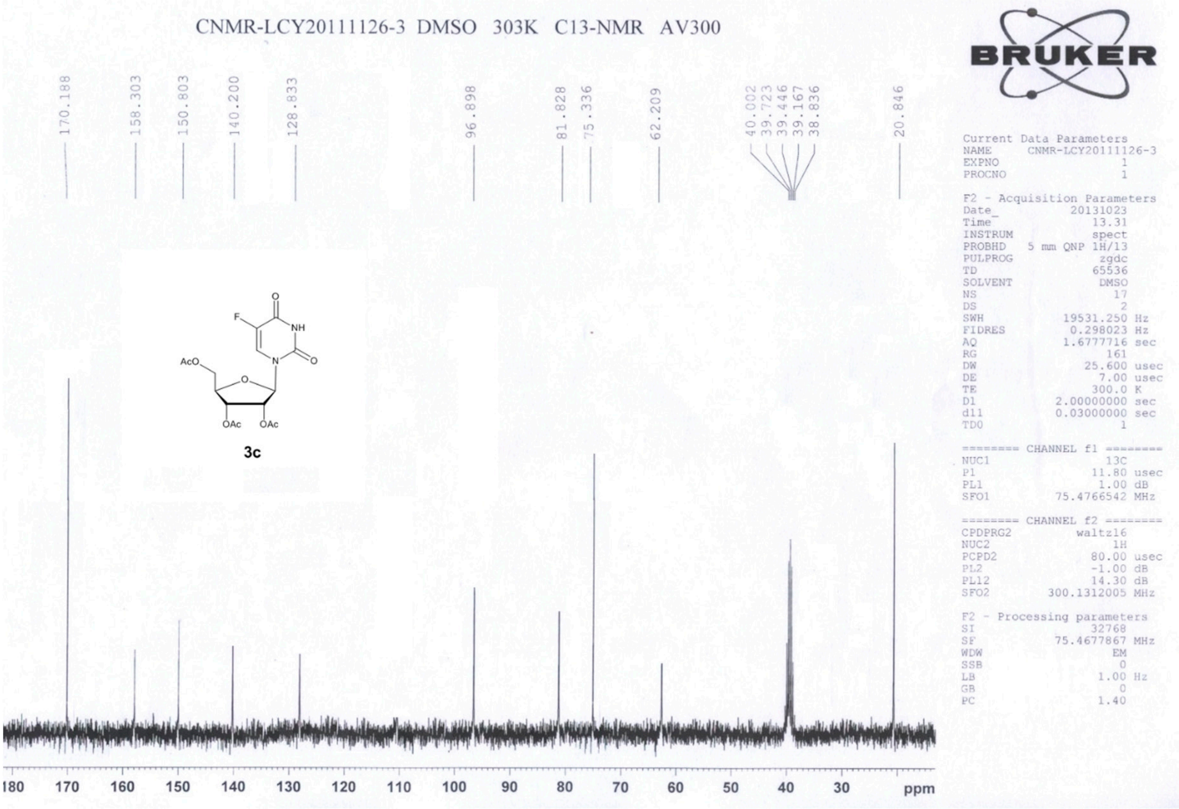

6.4. C13-NMR of **3d**

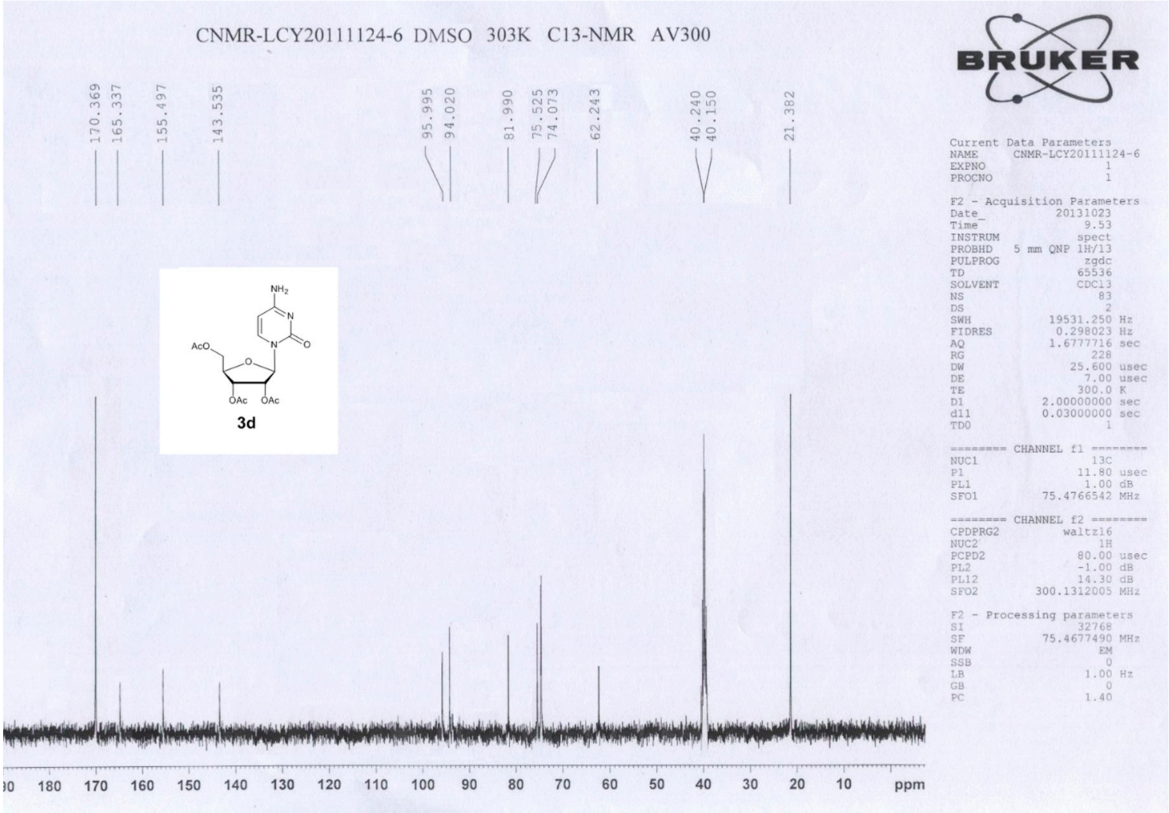

6.5. C13-NMR of **3e**

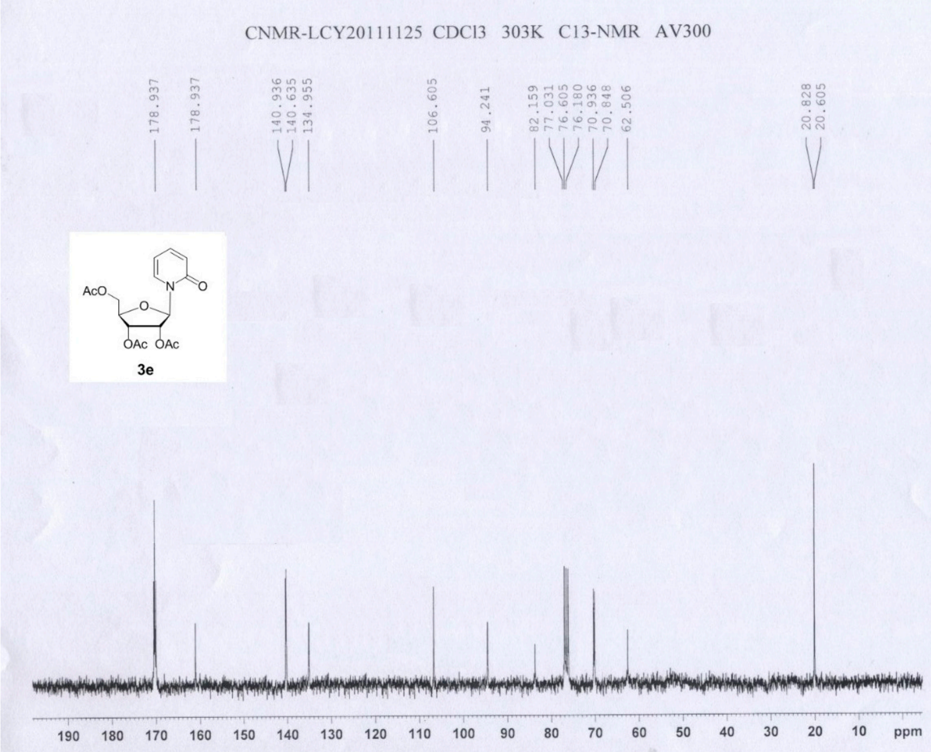

6.6. C[13]-NMR of **3f**

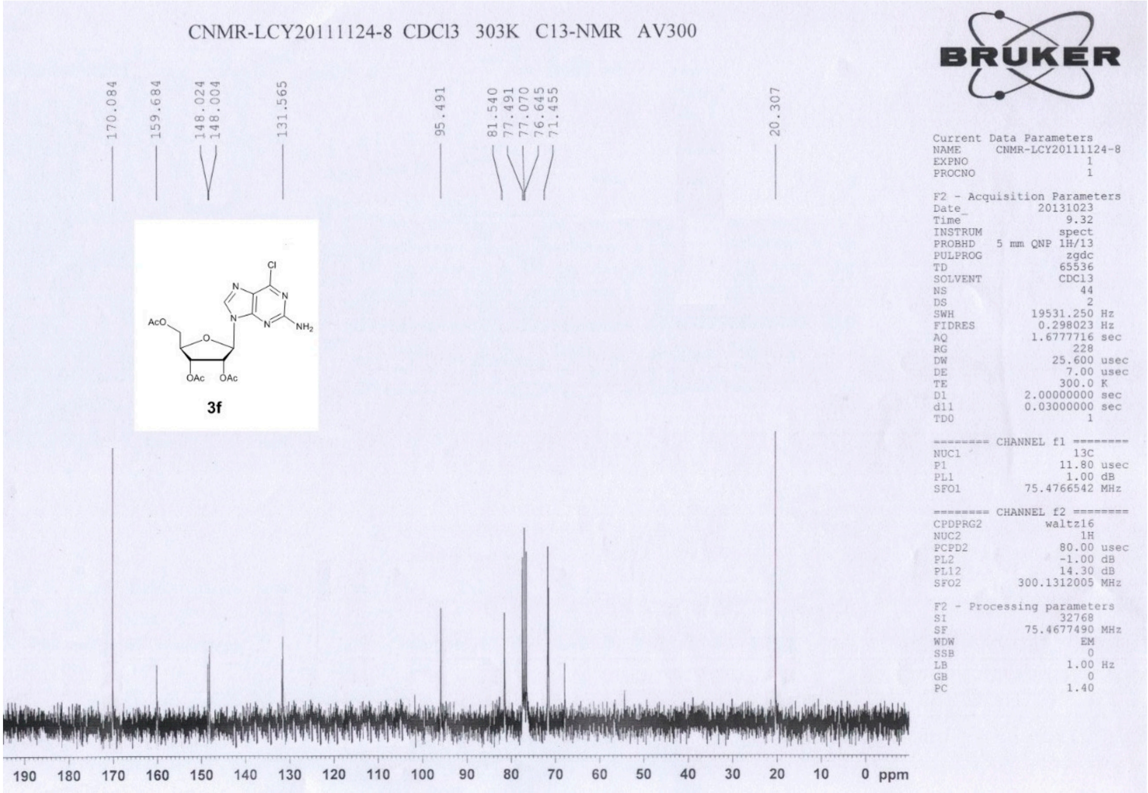

6.7. C[13]-NMR of **3g**

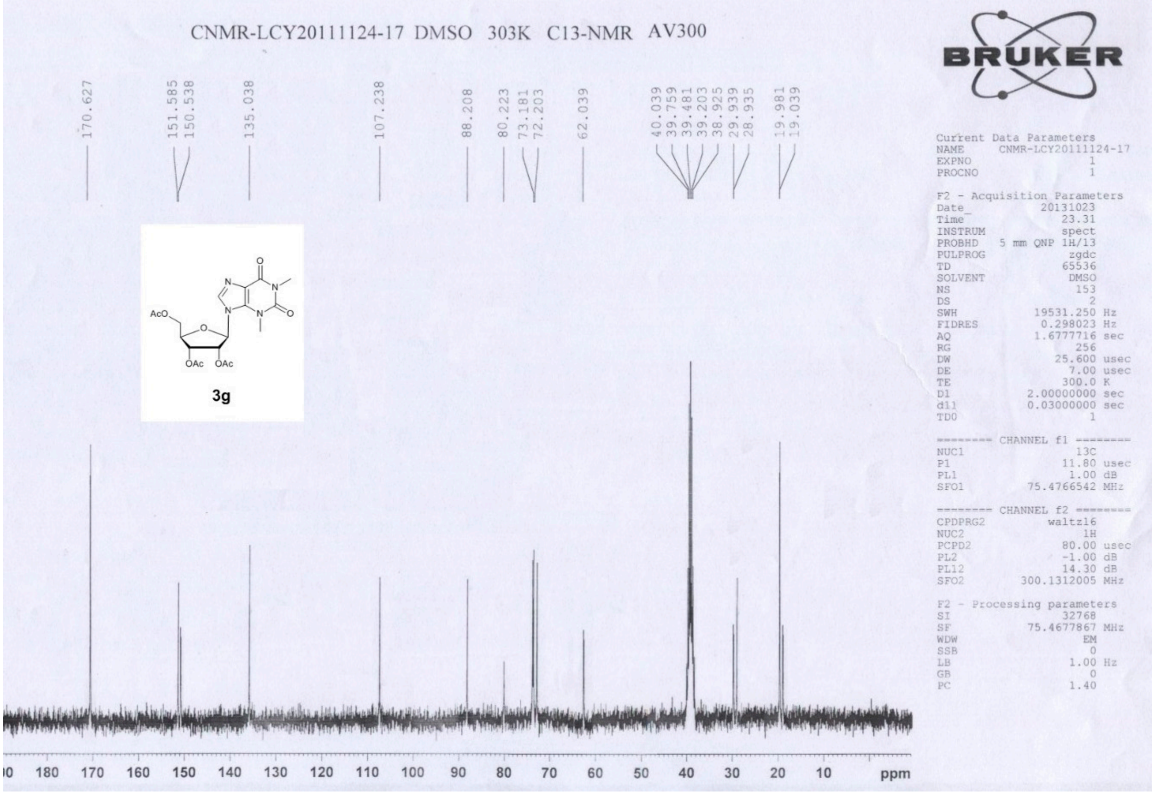

6.8. C[13]*-NMR of* ***3h***

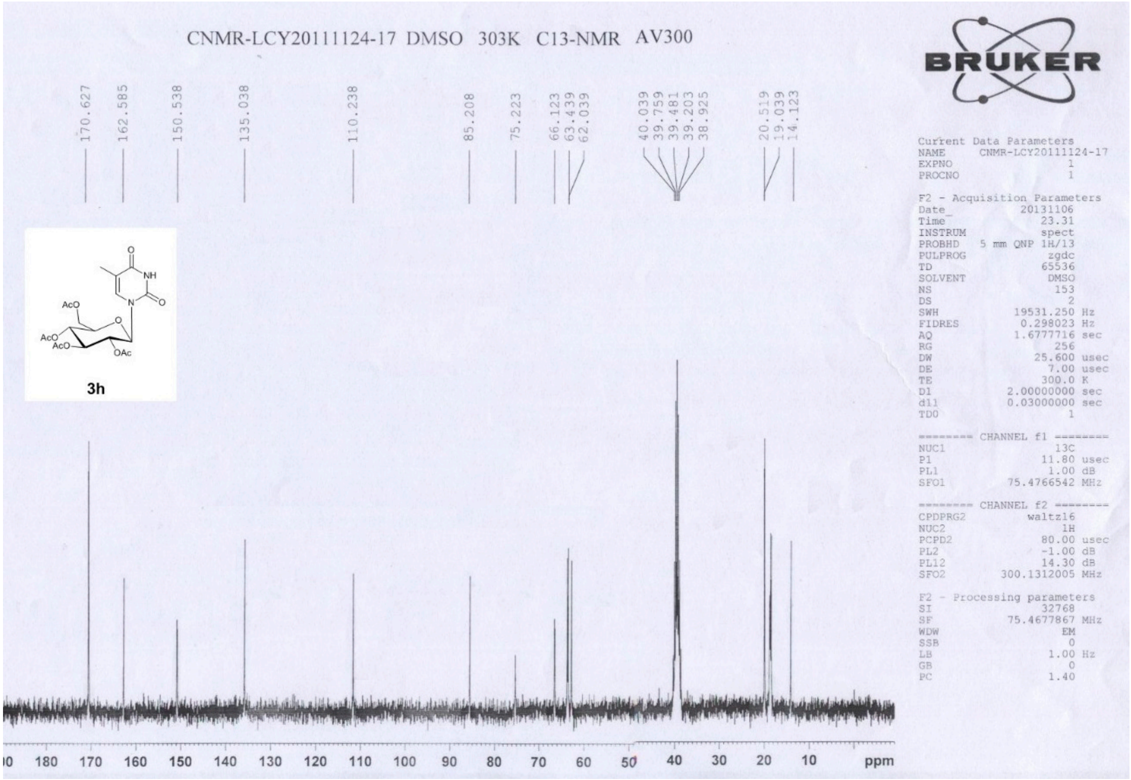

6.9. C[13]*-NMR of* ***3i***

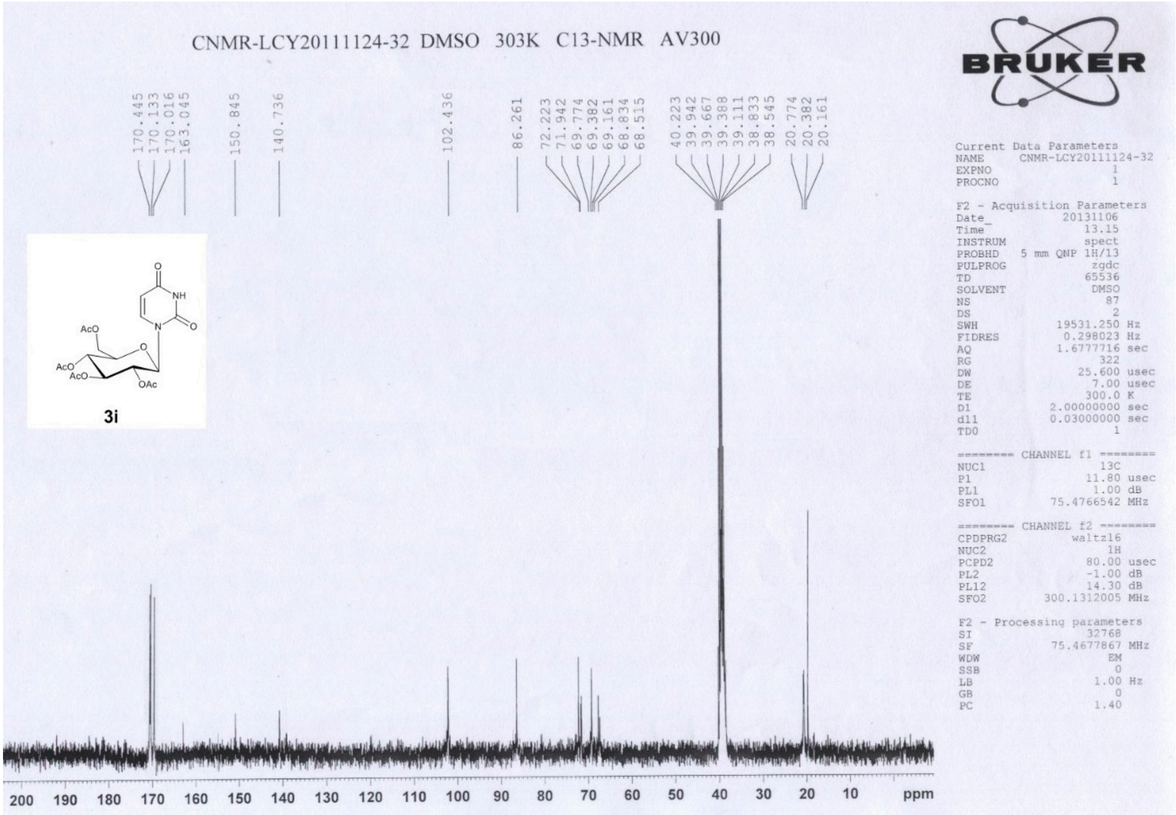

6.10. C[13]-NMR of ***3j***

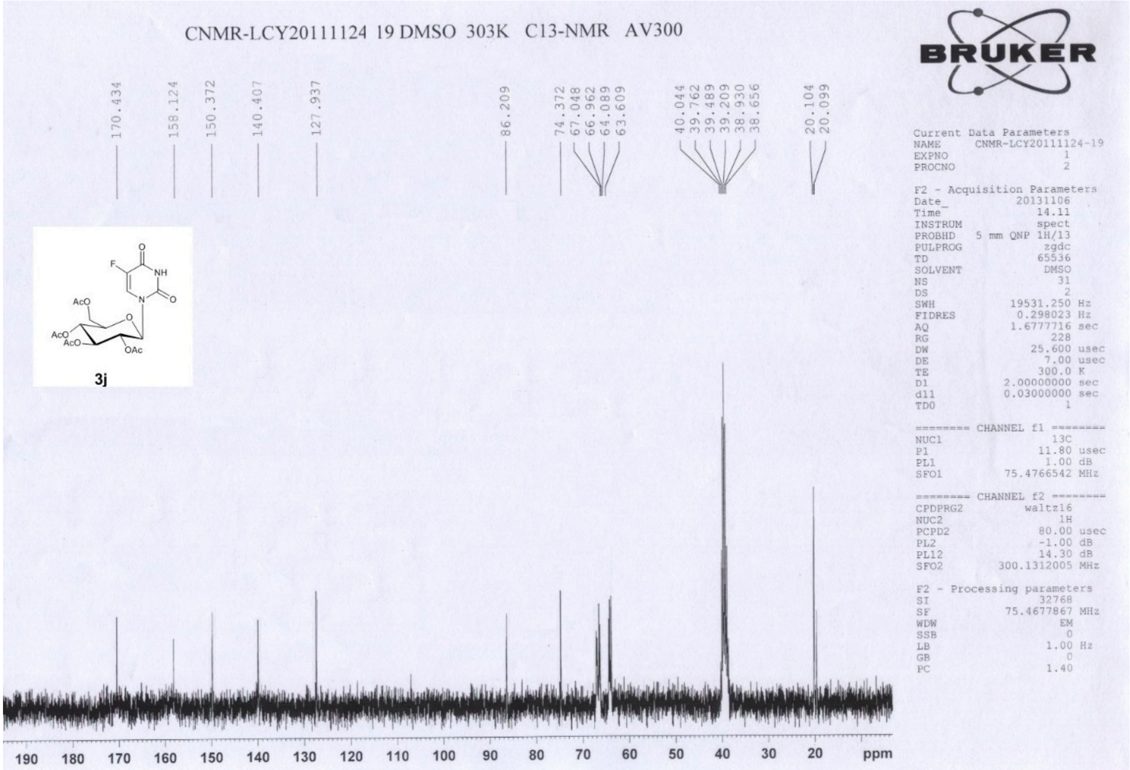

6.11. C[13]-NMR of ***3k***

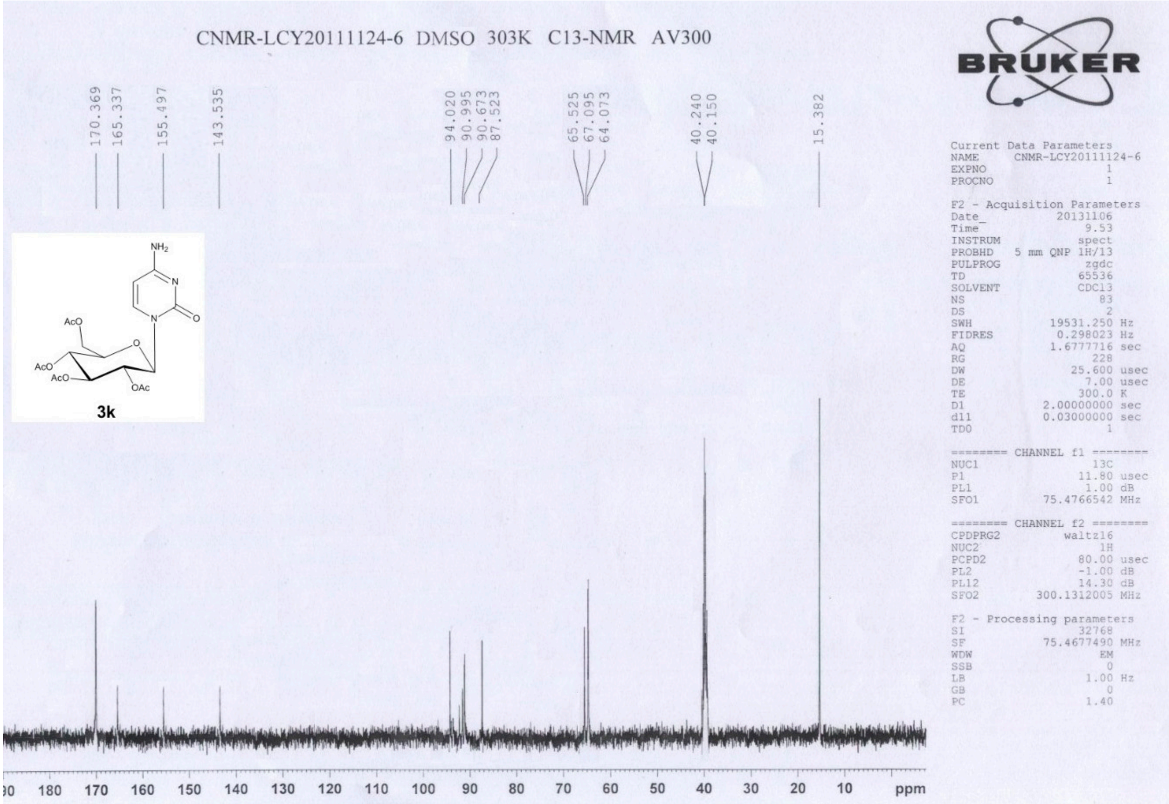

6.12. C[13]-NMR of **3l**

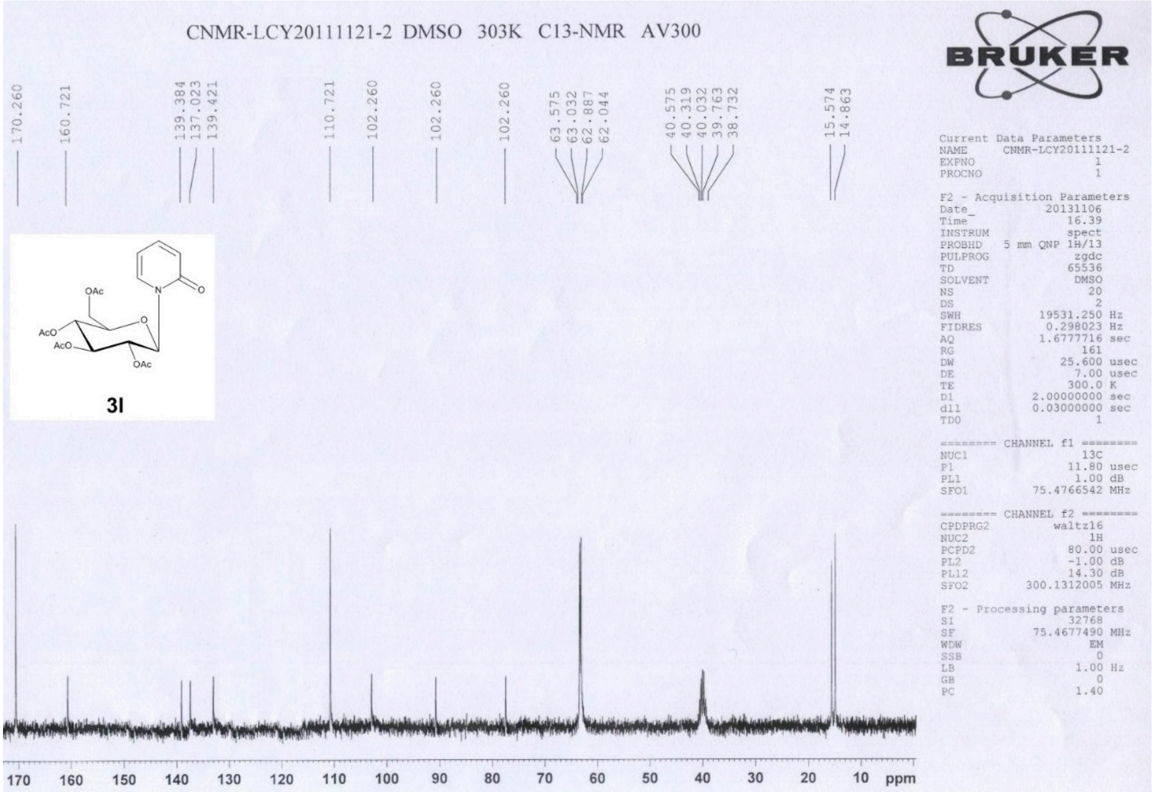

6.13. C[13]-NMR of Capecitabine

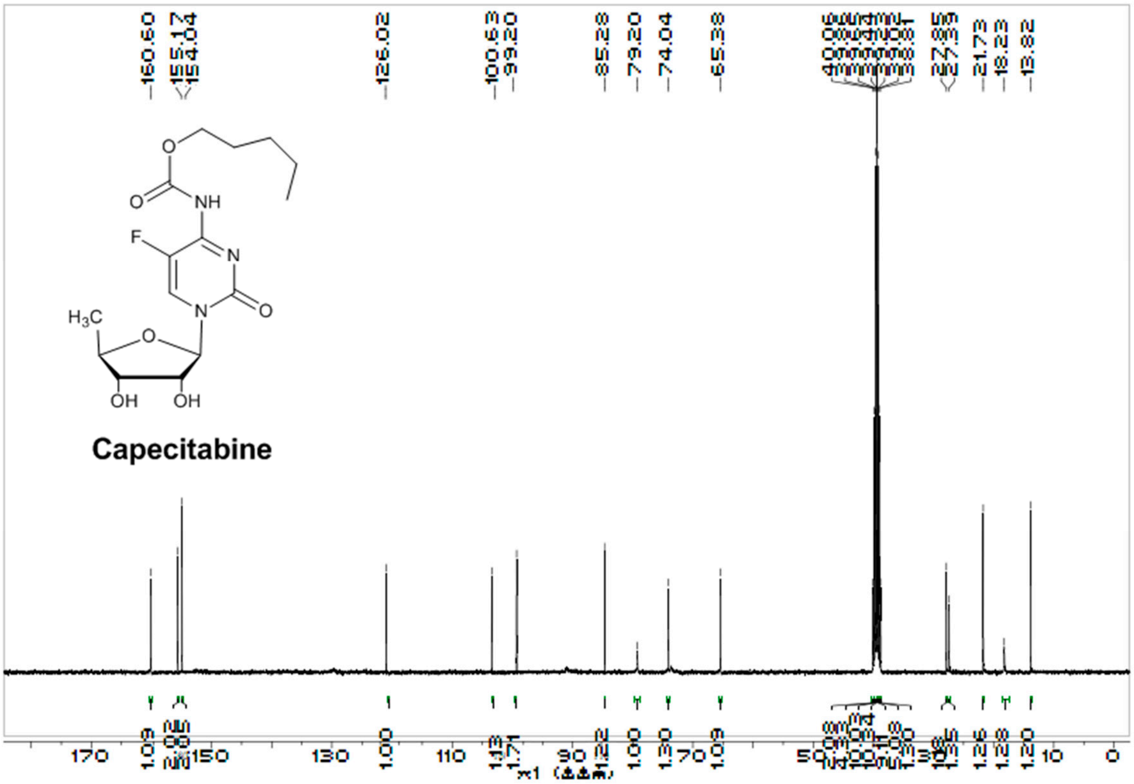

7. Mass Spectrometry

7.1. MS of **1a**

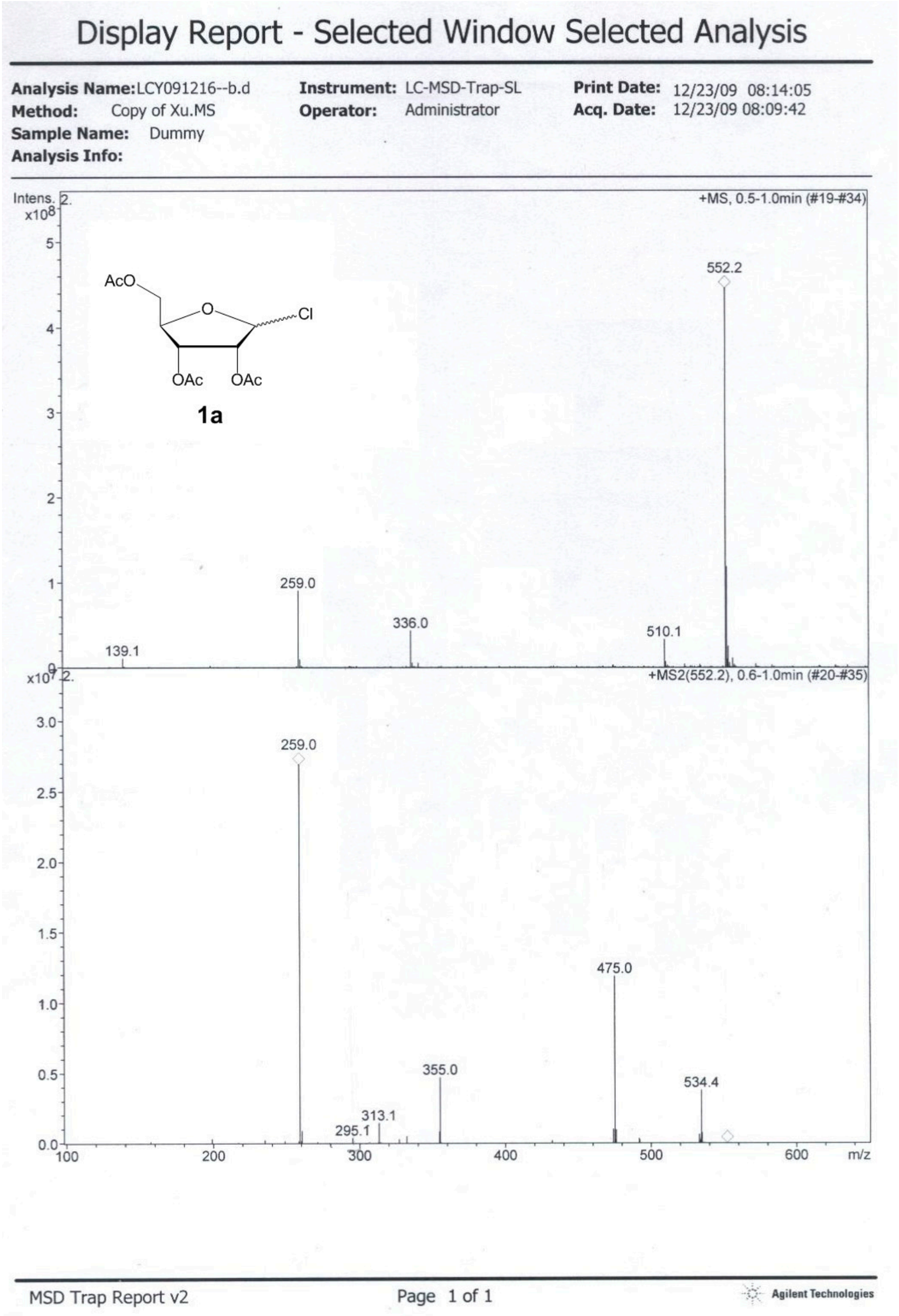

7.2. *MS of* **3a**

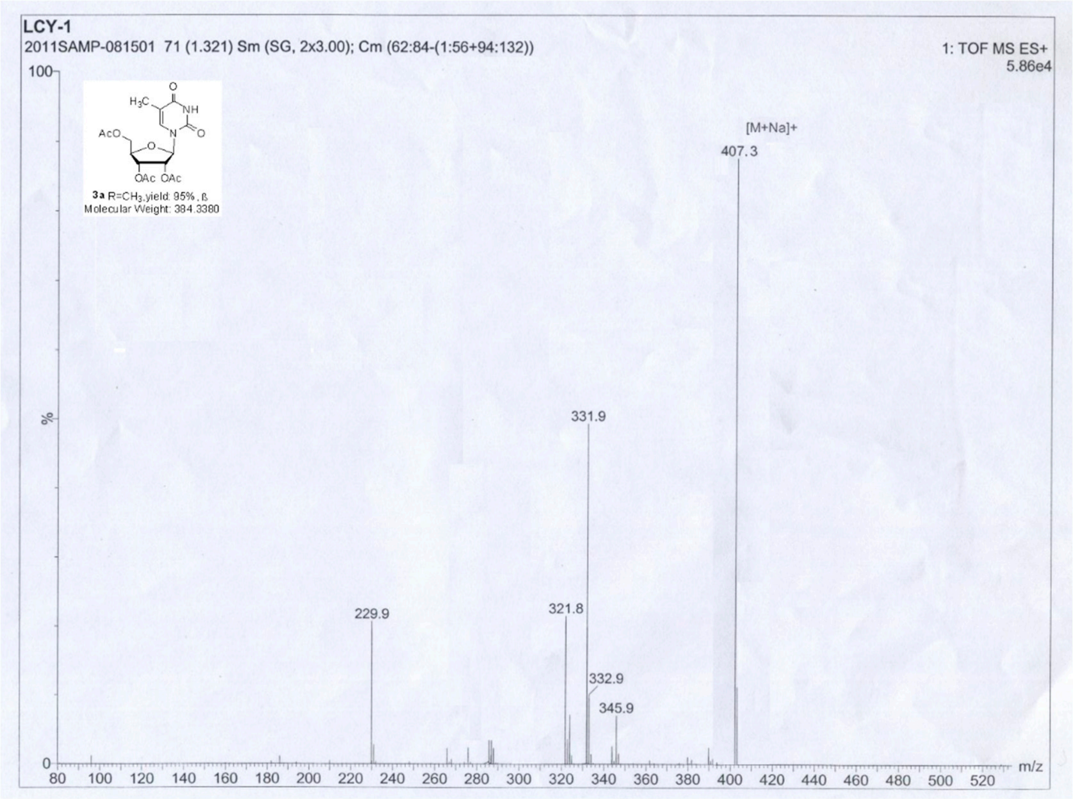

7.3. *MS of* **3b**

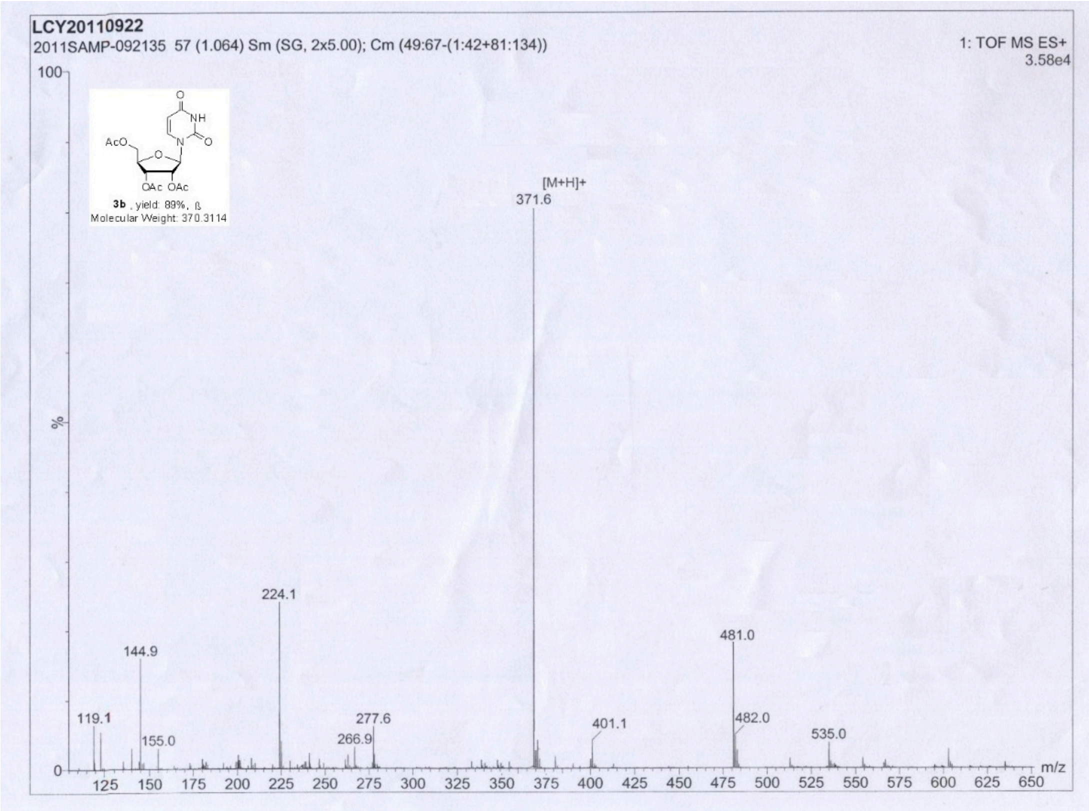

7.4. MS of **3c**

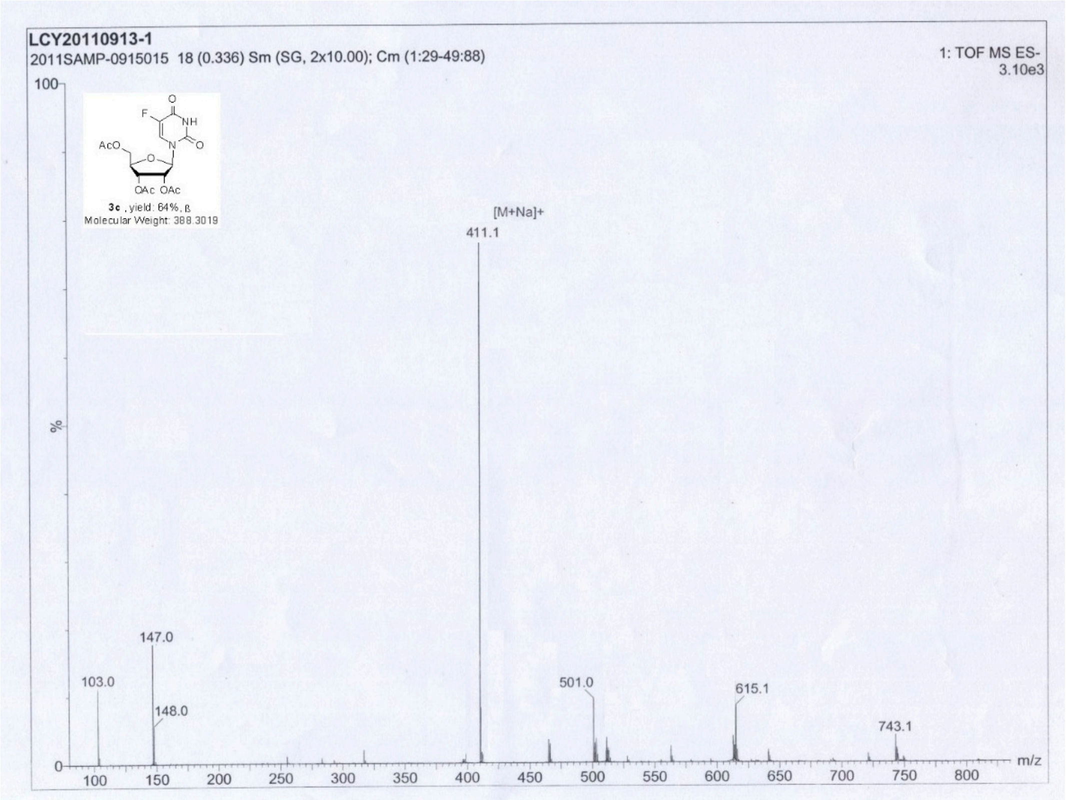

7.5. MS of **3d**

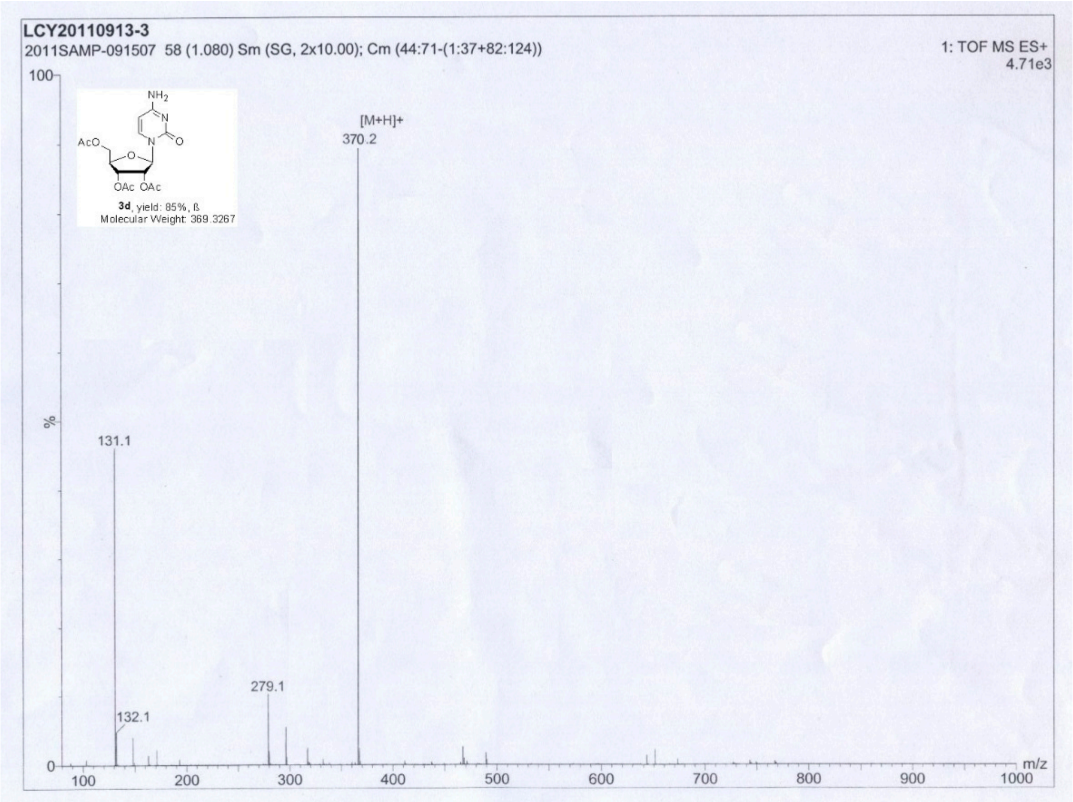

7.6. *MS of* **3e**

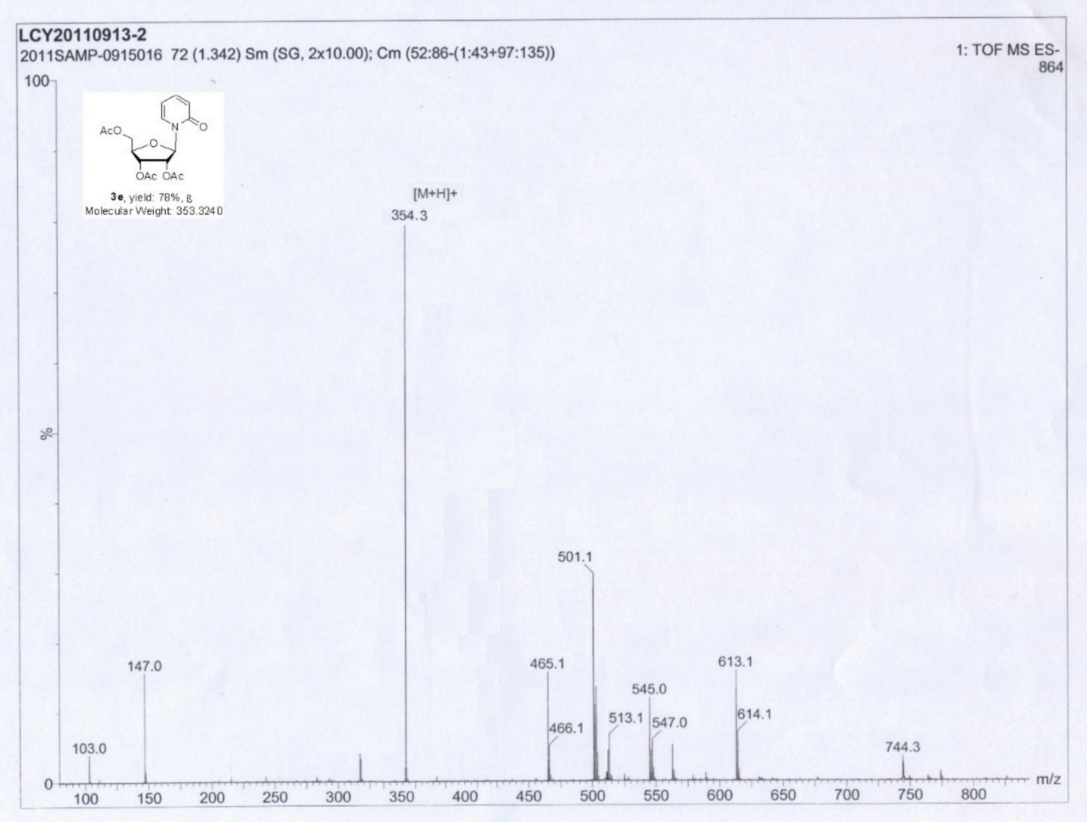

7.7. *MS of* **3f**

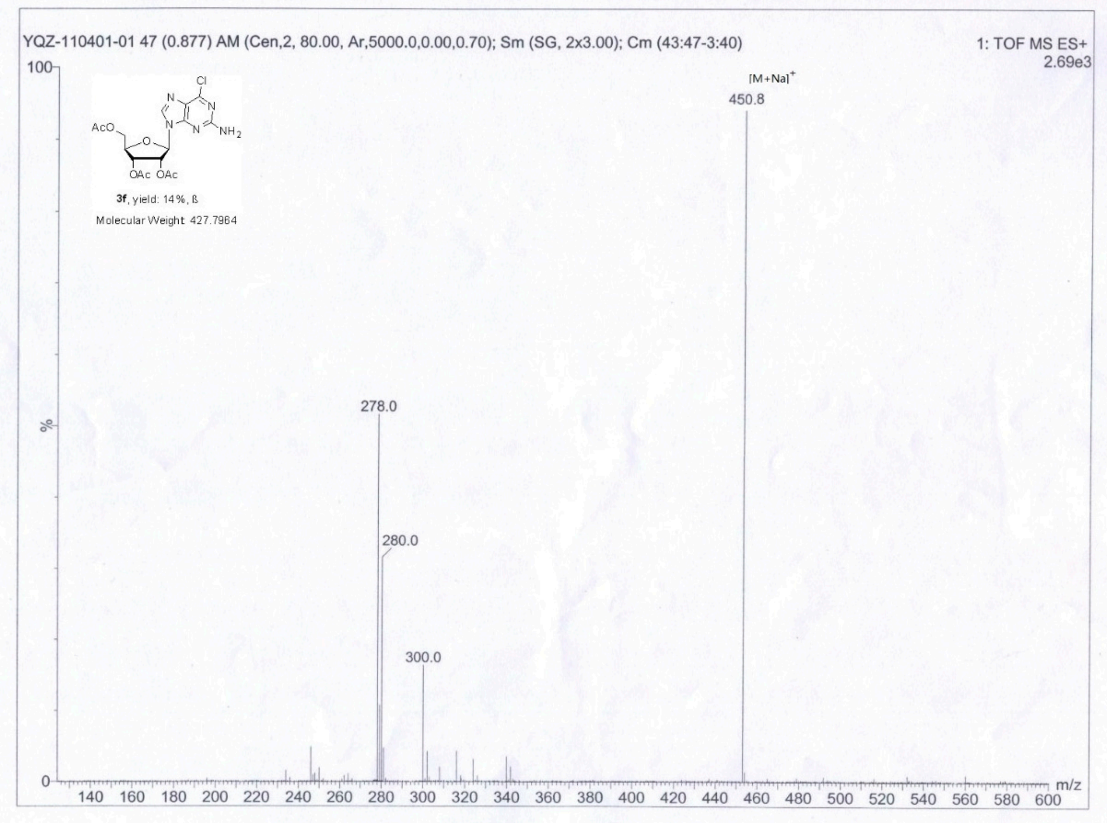

7.8. MS of **3g**

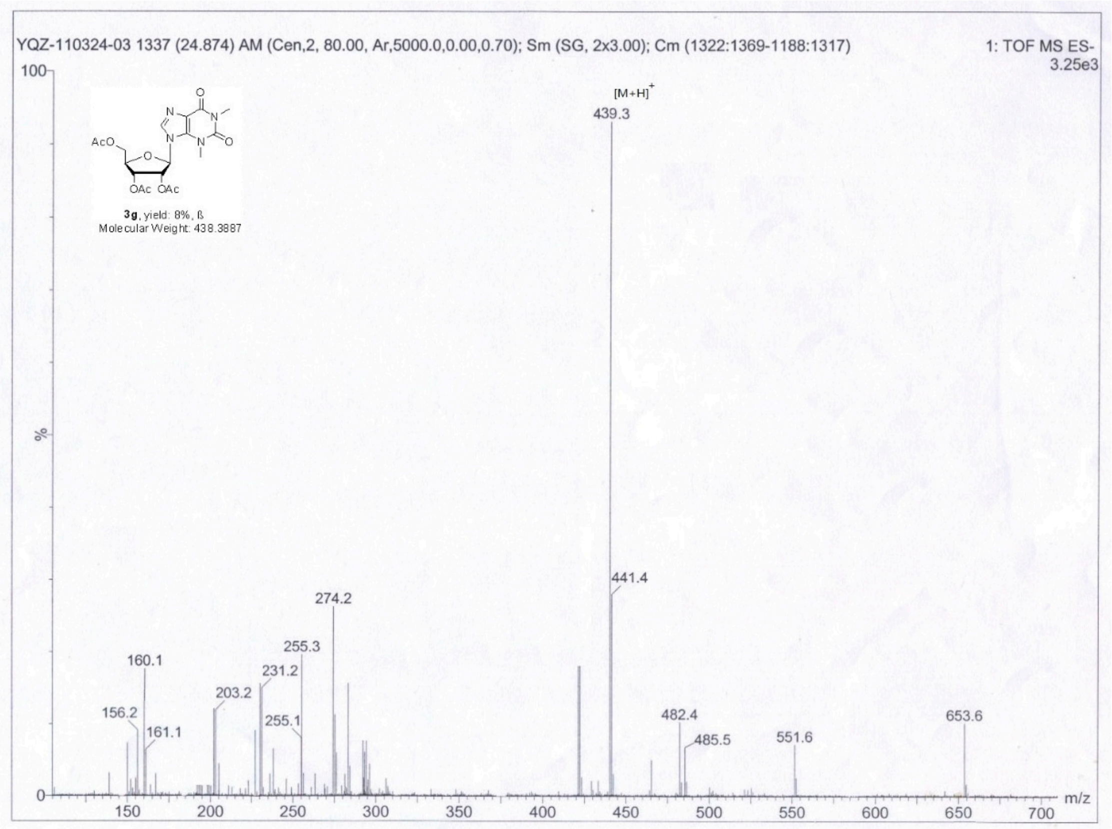

7.9. MS of **3h**

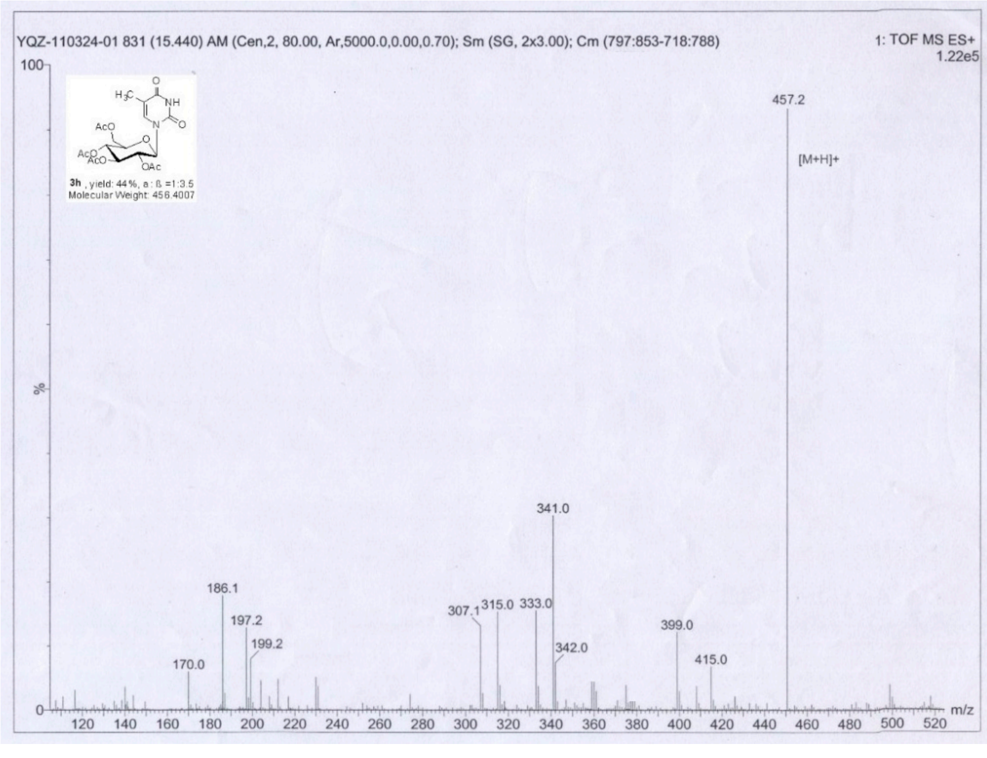

7.10. MS of **3i**

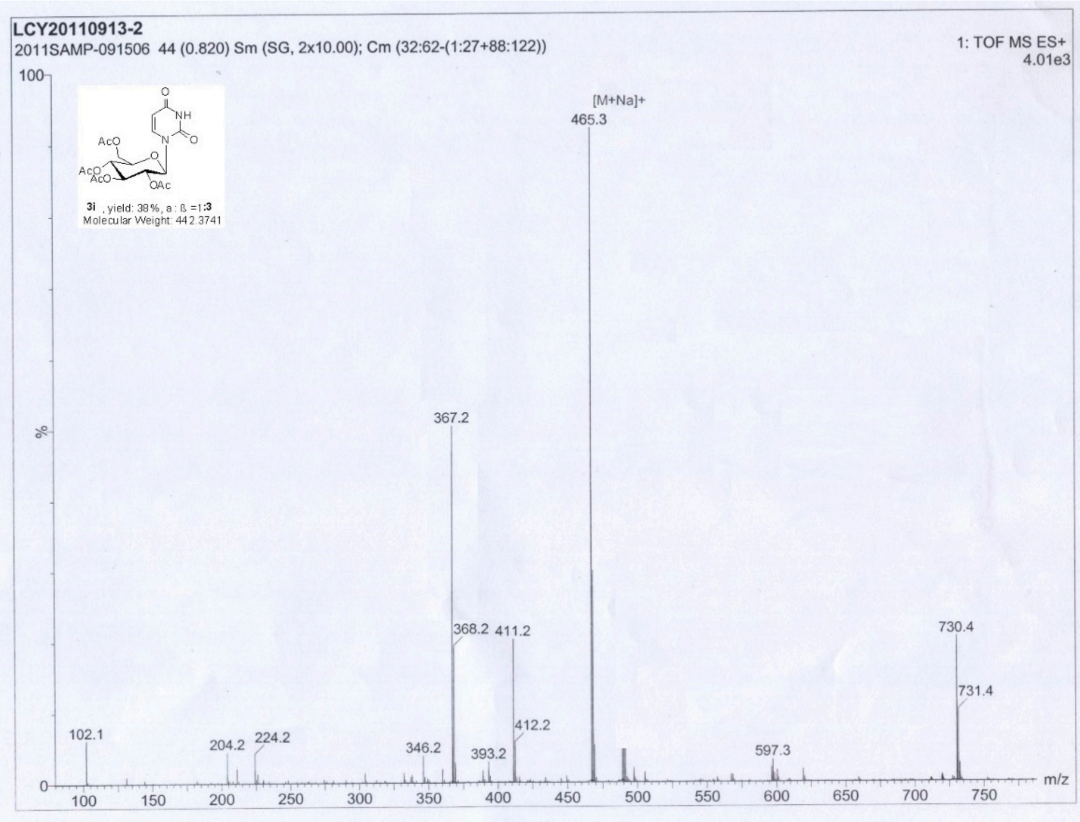

7.11. MS of **3j**

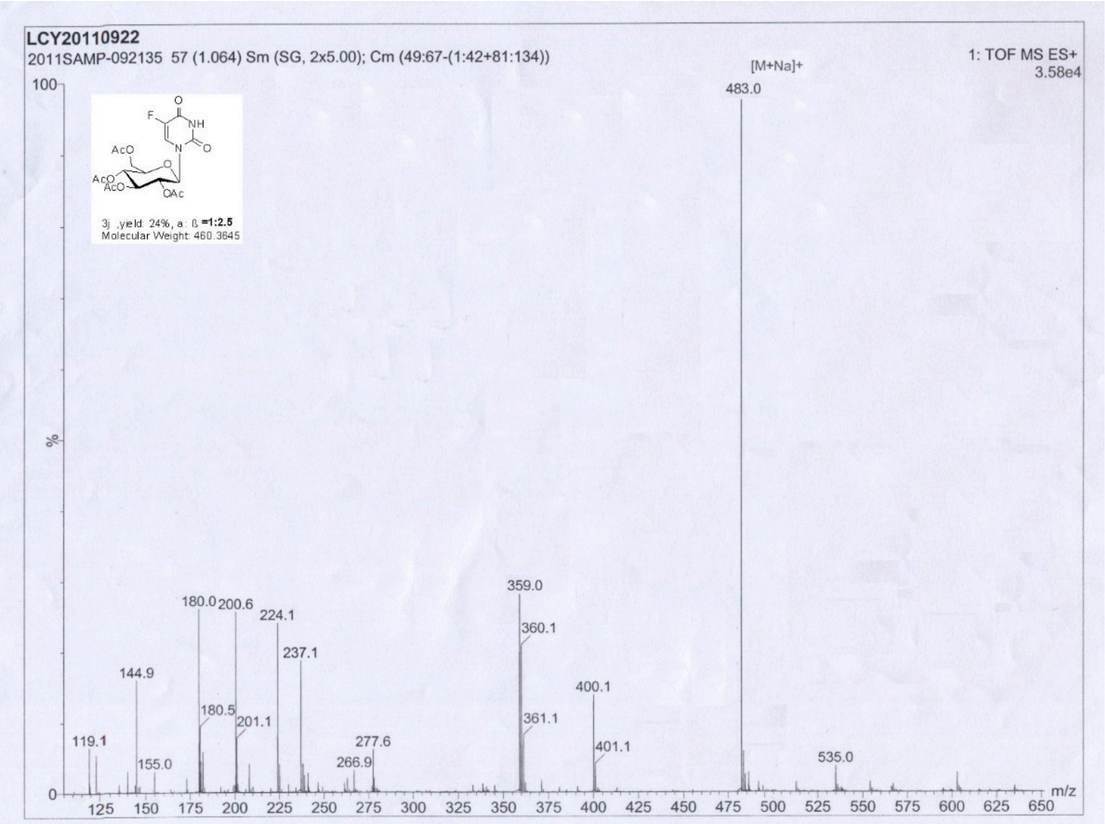

7.12. *MS of* **3k**

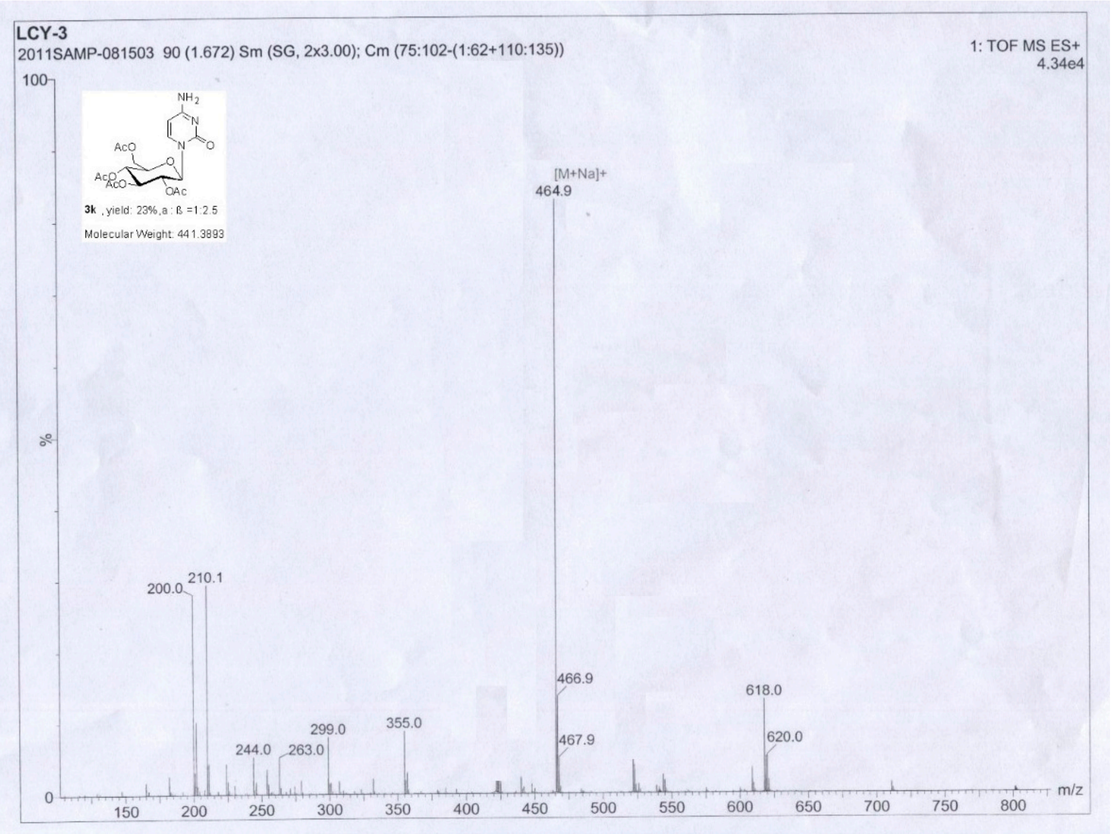

7.13. *MS of* **3l**

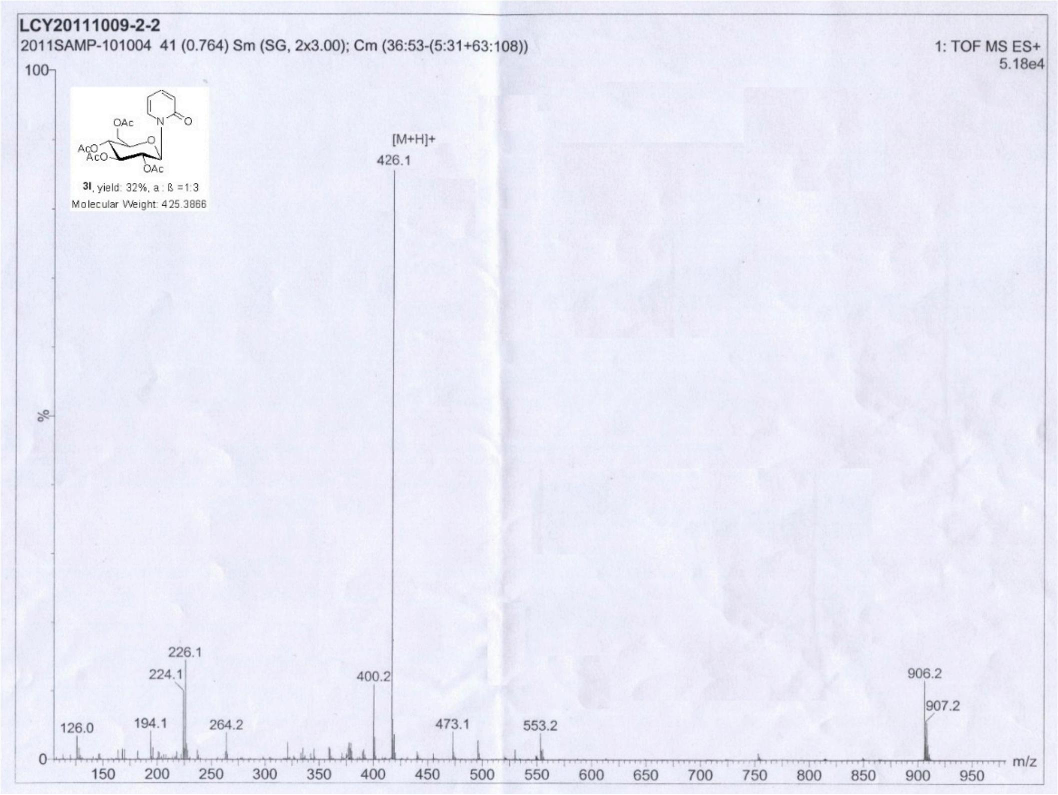

7.14. MS of Capecitabine

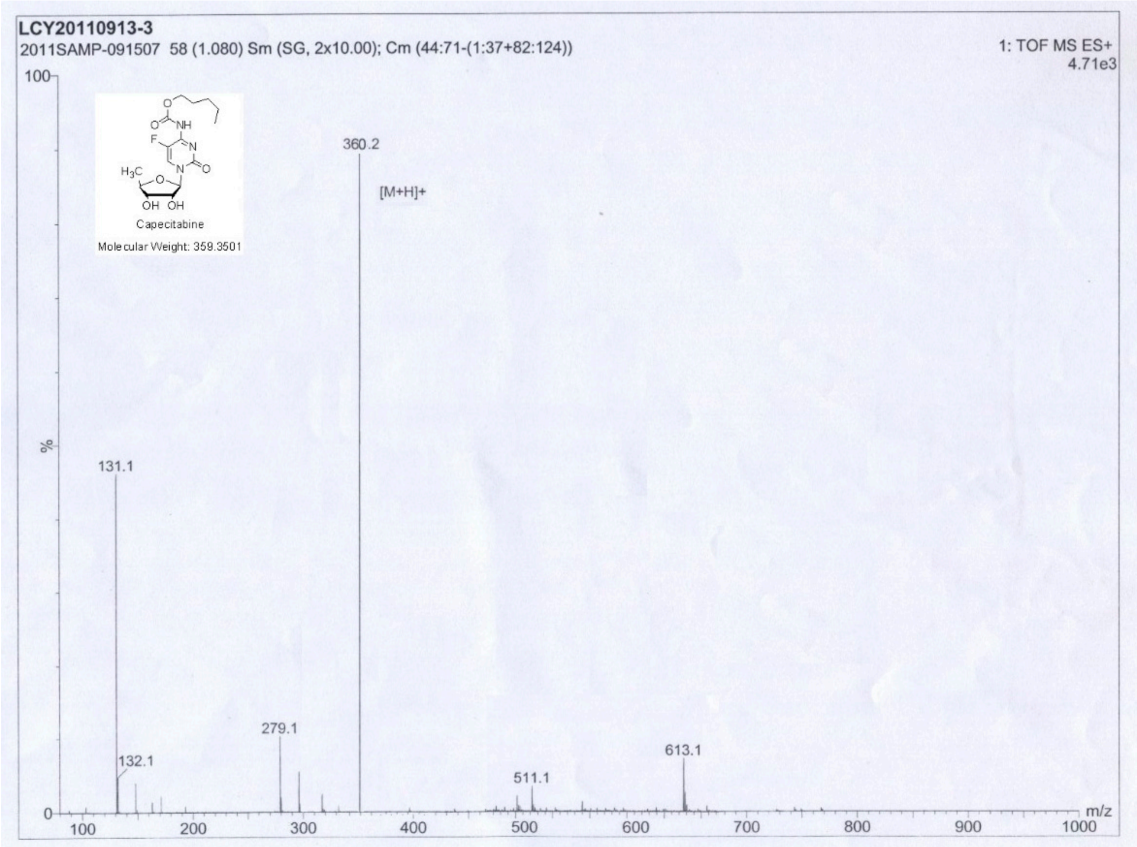

Supplement: Supplementary file 1 [file molecules-22-00084-s001.pdf]
